# Machine Learning and Molecular Modeling for Drug Repurposing Targeting Potential PI3Kα Inhibitors in Post-CoViD-19 Pulmonary Fibrosis

**DOI:** 10.1021/acsomega.5c08980

**Published:** 2026-01-01

**Authors:** Carine Ribeiro dos Santos, Priscila Goes Camargo, Carlos Rangel Rodrigues, Camilo Henrique da Silva Lima, Magaly Girão Albuquerque

**Affiliations:** † 28125 Universidade Federal do Rio de Janeiro (UFRJ), Centro de Ciências Matemáticas e da Natureza (CCMN), Instituto de Química (IQ), Departamento de Química Orgânica (DQO), Programa de Pós-Graduação em Química (PGQu), Laboratório de Modelagem Molecular Prof. Ricardo Bicca de Alencastro (LabMMol), Avenida Athos da Silveira Ramos, n° 149, Centro de Tecnologia, Bloco A, Cidade Universitária, Rio de Janeiro, Rio de Janeiro CEP 21941-909, Brazil; ‡ Universidade Federal do Rio de Janeiro (UFRJ), Centro de Ciências da Saúde (CCS), Faculdade de Farmácia (FF), Departamento de Fármacos e Medicamentos (DEFARMED), Laboratório de Modelagem Molecular & QSAR (ModMolQSAR), Avenida Carlos Chagas Filho, 373, Cidade Universitária, Rio de Janeiro, Rio de Janeiro CEP 21941-902, Brazil

## Abstract

Dysregulation of the phosphoinositide 3-kinase-alpha (PI3Kα) pathway is implicated in the development of post-CoViD-19 pulmonary fibrosis, highlighting the need for effective therapeutic agents. This study aimed to identify novel PI3Kα inhibitors by computationally repurposing FDA-approved drugs. We employed a hybrid approach that combines machine learning with molecular modeling. A random forest (RF) classification model was built and validated using a curated data set of 4,023 known PI3Kα inhibitors from the ChEMBL database, demonstrating robust predictive performance. The RF model was applied to screen the subset of FDA-approved drugs available in the DrugBank database to identify potential candidates. The top-ranked compounds were subsequently evaluated through molecular docking, extensive 200 ns molecular dynamics simulations (MDS), and binding free energy calculations using the molecular mechanics/Poisson–Boltzmann surface area (MM/PBSA) method. Our virtual screening identified five promising drugs, with simeprevir and ceritinib demonstrating the most favorable free energy binding affinities (Δ*G*_bind_ = −33.0 ± 3.2 kcal/mol and −25.2 ± 2.4 kcal/mol, respectively) and stable interactions within the enzyme’s kinase domain. These findings highlight simeprevir and ceritinib as strong candidates for PI3Kα inhibition, warranting further experimental investigation for their potential use in treating post-CoViD-19 fibrotic conditions.

## Graphical abstract

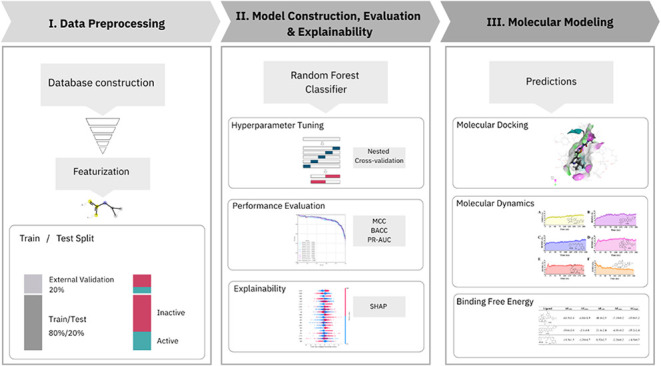

## Introduction

Phosphoinositide 3-kinases (PI3Ks) are a group of enzymes that play a crucial role in various cellular processes, including growth, proliferation, and survival.^1–3^ PI3Ks phosphorylate phosphatidylinositol (PI) and its phosphorylated derivatives to produce 3-phosphoinositides, allowing them to act as ligands and regulators of various proteins that activate essential intracellular signaling pathways.^3–5^ Class I PI3K enzymes are heterodimers, composed of a p110 catalytic subunit (isoforms α, β, γ, and δ) complexed to a p85 regulatory subunit, responsible for converting phosphatidylinositol-(4,5)­bisphosphate (PIP2) into phosphatidylinositol-(3,4,5)-triphosphate (PIP3),^2,6,7^ which in turn activates protein kinase B (PKB, also known as AKT) and other effectors to regulate multiple cellular functions.^8^ Disorders in the PI3K I metabolic pathway are associated with different diseases, with mutations in the p110α isoform (PI3Kα), encoded by the PIK3CA gene, being linked to breast, colon, and lung cancers.^7,8^ Dysregulation of this pathway also has implications for other diseases, such as diabetes and cardiovascular diseases.^9^

The CoViD-19 pandemic has brought attention to the importance of PI3K, as its impact on the inflammatory response can lead to excessive scar tissue formation in the lungs, resulting in pulmonary fibrosis.^10^ This condition occurs due to the disruption of inflammatory and pro-fibrotic signaling pathways, including the PI3K/AKT pathway, which triggers epithelial–mesenchymal transition (EMT) and extracellular matrix (ECM) deposits.^11^ Research suggests that regulating PI3K activity, especially the α isoform, could be beneficial in preventing and treating post-CoViD-19 pulmonary fibrosis.^12–15^

While a definitive link between CoViD-19 and lung cancer remains speculative, mechanistic hypotheses are under investigation. These center on virus-induced chronic inflammation (IL-6/JAK/STAT3), genomic instability from impaired DNA repair and suppression of p53/PRB, and chronic endoplasmic reticulum (ER) stress. Critically, the viral downregulation of the ACE2 receptor is theorized to activate pro-tumorigenic signaling cascades, including the PI3K/AKT pathway.^16^

Mechanistically, the PI3K/AKT pathway functions as a critical convergent point for multiple pro-fibrotic signaling cascades. Its inhibition is hypothesized to attenuate fibrosis through several key processes^17^ (Figure 1). First, it interrupts the noncanonical (non-SMAD) signaling of transforming growth factor-β (TGF-β), the primary mediator of fibrosis, which utilizes the PI3K/AKT pathway to sustain the fibrotic response. Second, the pathway is a major downstream effector for other key pro-fibrotic stimuli, including platelet-derived growth factor (PDGF) and fibroblast growth factor (FGF). These growth factors bind to their respective receptors (PDGFR/FGFR) on fibroblasts, triggering the activation of PI3K. Once activated, Class I PI3Ks phosphorylate PIP2 to generate PIP3, a step that is negatively regulated by the phosphatase and tensin homologue (PTEN). This, in turn, activates AKT, which then activates its own downstream targets, such as mTOR and HIF-1a that inhibits autophagy and maintains the high proliferation and antiapoptosis characteristics of fibroblasts, upregulating the production of collagens I and III, the main components of excessive ECM deposition (Figure 1).^18^

**Figure 1:**
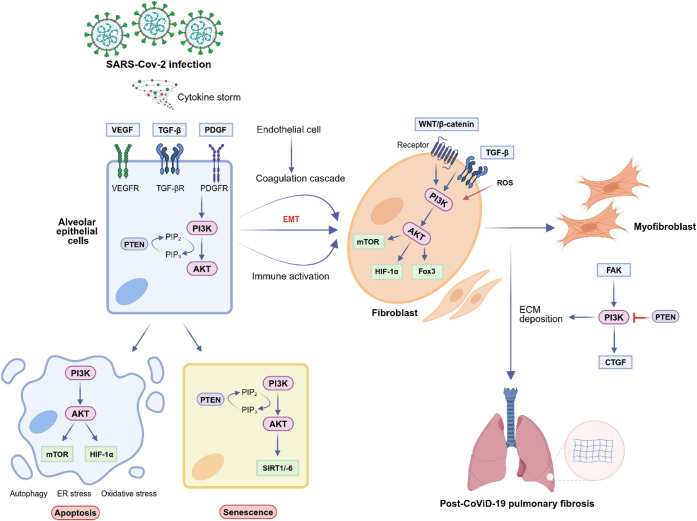
Schematic illustration of the pathways involved in pulmonary fibrosis following SARS-CoV-2 infection. It highlights the roles of alveolar epithelial cells, endothelial cells, and myofibroblasts in the immune activation and coagulation cascade. Key signaling molecules, including VEGF, TGF-β, and WNT/β-catenin, are indicated to mediate fibroblast activation and extracellular matrix deposition. Processes such as apoptosis and senescence are shown to contribute to the disease’s progression. Created in BioRender. Ribeiro dos Santos (2025) https://BioRender.com/m6i37mt.

A distinct pathway involving focal adhesion kinase (FAK) contributes to the fibrotic outcome (Figure 1). FAK, often activated by integrin signaling from a stiff ECM, activates PI3K. This FAK-mediated activation leads to critical downstream effects, including the inhibition of PTEN, which creates a feed-forward loop to amplify and sustain PI3K signaling, and the upregulation of connective tissue growth factor (CTGF) that promotes excessive ECM deposition and tissue remodeling.^18^

This PI3K/AKT/mTOR axis is a driver of the fibrogenic process, directly regulating the cellular processes that define fibrosis such as fibroblast proliferation, motility, and survival. In particular, the PI3Kα isoform is frequently upregulated in lung-related diseases and plays a major role in sustaining the TGF-β-induced increase in proliferation. Given this, the mechanistic rationale for inhibiting this is to directly disrupt these core fibrotic processes. Inhibition is expected to attenuate EMT, reduce fibroblast proliferation and survival, and suppress the differentiation of myofibroblasts, thereby limiting the excessive ECM deposition that leads to pulmonary fibrosis (Figure 1).^17,19^

In 2019, the Food and Drug Administration (FDA) approved alpelisib, a potent selective ATP-competitive inhibitor of PI3Kα, for treating patients with severe PIK3CA-related overgrowth spectrum (PROS), i.e., a group of diverse overgrowth disorders caused by PIK3CA mutations.^9^ Additionally, several compounds were analyzed focusing on the PI3K/AKT pathway for idiopathic pulmonary fibrosis (IPF)^18,20^ and post-CoViD-19 pulmonary fibrosis (PC19-PF or PCPF)^13,21–23^ due to similarities between IPF and PCPF.^14,24^

Several PI3K inhibitors, including omipalisib, HEC68498, duvelisib, and rapamycin, have been repurposed for IPF. In preclinical models, omipalisib was shown to reduce fibroblast proliferation and collagen synthesis.^25^ A phase I clinical trial (NCT01725139) indicated that omipalisib was safe and demonstrated dose-dependent inhibition of the PI3K/mTOR pathway in individuals with IPF. Similarly, other inhibitors such as PX-866, duvelisib, and LY294002 have exhibited antifibrotic effects in animal models of pulmonary fibrosis, suggesting the pathway’s relevance in disease pathology. Despite these findings, substantial challenges like incomplete clinical data, unresolved mechanisms of action, and safety concerns impede the clinical application of these compounds, indicating a need for further research.^26^

The research and development (R&D) of a new drug is a costly, time-consuming process that can span over a decade.^27^ Computer-aided drug design (CADD) offers a more efficient and cost-effective approach.^28–31^ CADD encompasses two main strategies: ligand-based drug design (LBDD), which relies on the properties of known active molecules, and structure-based drug design (SBDD), which utilizes the 3D structure of the biological target.^32^

Computational techniques, such as machine learning (ML) in an LBDD approach, in combination with molecular docking and molecular dynamics simulations, in an SBDD approach, are widely used to identify and optimize new drug candidates.^33–36^ Machine learning and deep learning, subareas of artificial intelligence, aid in finding patterns within data sets to create predictive models for searching bioactive compounds.^37^

Molecular docking is a computational modeling technique that predicts the preferred conformation and orientation of a ligand within the binding site of a receptor. By combining search algorithms and scoring functions, the method evaluates poses in a computationally efficient manner to estimate the binding affinity. Frequently, its output serves as a starting point for more rigorous methods, selecting the most probable poses to be subsequently analyzed in molecular dynamics simulations (MDS), which investigate the stability and dynamics of the complex.^38^

Molecular dynamics simulation studies allow the evaluation of interactions between small ligands and their target proteins, providing a detailed view of their atomic and conformational movements, which are time- and solvation-dependent.^29,39,40^ The affinity between ligands and their target proteins obtained by MDS is more precise than that obtained by molecular docking, enabling a closer correlation with biological activity obtained experimentally.^33,34,41^ Several works have been carried out using these computational techniques to understand the mechanism of action of known inhibitors or to search for new ones for the PI3K/AKT pathway or specifically for PI3K.^42–45^

It is essential to highlight the rheumatoid arthritis drug baricitinib that was repurposed for the treatment of CoViD-19 by using artificial intelligence methods,^46^ showing the versatility of the CADD approach in the search for new drugs.^47,48^ Therefore, this repurposing study aims to identify potential inhibitors of the PI3Kα enzyme as new drug candidates from a DrugBank/FDA-approved drugs data set, utilizing computational techniques, including machine learning, molecular docking, and molecular dynamics simulations. These findings could be applied in the treatment of post-CoViD-19 pulmonary fibrosis.

## Materials and Methods

### Data Set Selection and Curation

Considering the *Homo sapiens* PI3K p110α catalytic subunit (*Hs*PI3Kα) as the biomolecule target, the *Hs*PI3Kα data set (i.e., chemical structures and biological activities) used in this study was collected from the ChEMBL (v.34) database of bioactive drug-like molecules (https://www.ebi.ac.uk/chembl/)^49^ under the ChEMBL ID ChEMBL4005 and corresponds to a total of 12269 compounds. Of this total, 6876 compounds have activity values recorded in half-maximal inhibitory concentration (IC_50_, nM) which were converted to −Log IC_50_ (pIC_50_, M). Compounds with null or invalid (non-numeric) values of IC_50_, mutations, and/or assay types different from “binding” (binding is 99% majority) were removed from the data set to keep the data uniform.

The ChEMBL Pipeline^50^ was used for chemical structure curation in order to standardize all chemical structures by removing mixtures and nonorganic compounds (e.g., inorganics, counterions, metals, and organometallics), and normalizing specific chemotypes (e.g., hypervalent nitro groups, amide tautomers, triple bonds, and allenes).^50^ Morgan (circular) fingerprints^51,52^ were employed within the RDKit software package^53^ to compute structural topological similarity by the Tanimoto algorithm^54^ and eliminate duplicates.

In cases where molecules exhibited 100% similarity but varied pIC_50_ values, we determined the mean value to represent the final pIC_50_. In our study, the compounds were ordered by pIC_50_ values, then the top 25% pIC_50_ compounds (highest pIC_50_ values) were labeled as active (positive, i.e., 1) PI3Kα inhibitors, with the subsequent 25% considered as intermediate compounds, and the remaining were labeled as inactive (negative, i.e., 0) PI3Kα inhibitors. Following the elimination of the intermediate compounds, a data set comprising 4023 compounds was acquired, consisting of 1354 active (∼34%, 7.79 ≥ pIC_50_ ≥ 9.82) and 2669 inactive (∼66%, 3.67 ≥ pIC_50_ ≥ 6.37) compounds.

### Descriptors Calculation: Molecular Fingerprints

The RDKit software package^53^ was utilized to compute the descriptors of the compounds in the data set. In this study, Morgan circular fingerprints^51,52^ with radius 2 (which includes radii 0, 1, and 2) and 2048 bits were used as molecular descriptors to describe each structure. Collinearity occurs when a pair of descriptors shows strong intercorrelation, which can increase model complexity and introduce potential bias.^55^

To address this issue, we calculated a pairwise correlation matrix in Python, using Pearson’s correlation coefficient (*r*) that measures the strength of the linear relationship between each pair of descriptors, where the *r* values range from −1 (total negative correlation) to +1 (total positive correlation), when two descriptors show *r* > |0.7|, one of the pair was filtered. We also removed descriptors with low variance, as they do not contribute much to distinguishing between compounds,^56^ with scikit-learn’s *VarianceThreshold* method, removing features with Var­[*X*] ≤ 0.16 (*p* = 0.8). This process results in a reduced subset of descriptors, which helps streamline the feature set, ensuring that the model remains efficient and interpretable.

### Data Set MODelability Index (MODI)

The MODelability Index (MODI) (eq 1)^57^ of the data set was calculated to evaluate the potential for developing predictive QSAR models for a binary data collection of bioactive substances. A data set is deemed suitable for modeling when the MODI surpasses a predefined threshold of 0.65,^61^ indicating a low fraction of activity cliffs, i.e., pairs of compounds that are highly similar (based on the smallest Euclidean distance), but have opposite activities, such as in the case of binary classification (e.g., active/inactive).^57^

1 $$\mathrm{MODI}=\frac{1}{K}\overset{K}{\underset{i=1}{\sum }}\frac{N_{i}^{\mathrm{same}}}{N_{i}^{\mathrm{total}}}$$

In eq 1, *K* is the number of classes (*K* = 2 for binary data sets), $N_{i}^{\mathrm{same}}$ is the number of compounds of *i*th activity class that have their first nearest neighbors belonging to the same activity class *i*, and $N_{i}^{\mathrm{total}}$ is the total number of compounds belonging to class *i*.^57^

### Model Choice

The LazyPredict^58^ (v.0.2.16) Python library was employed to perform rapid benchmarking of multiple machine learning algorithms using the same training data and preprocessing conditions. This tool provides a consistent framework for preliminary model comparison by automatically fitting and evaluating a broad set of classifiers, allowing for the identification of algorithms that yield the best baseline performance.

The random forest (RF) algorithm is a supervised machine learning technique that constructs an ensemble of decision trees by employing random subsets of data.^59^ It effectively mitigates issues related to overfitting through the utilization of multiple trees and the implementation of strategies such as bagging.^59,60^

Throughout the training phase, random samples extracted from the data set are used to train individual trees. Predictions are then generated by averaging the outputs of these trees or by a majority of votes in classification tasks. This method reduces model variance without introducing additional bias, thereby enhancing predictive capabilities.^59^

### Nested Cross-Validation

The scikit-learn^61^ Python package was used to split the original data into two, an internal set comprising 80% of the data, used for training and hyperparameter calculations in a nested cross-validation^62,63^ procedure, and an external set with the remaining 20%, used for the final validation of the model. This approach ensures that our model undergoes training and validation on distinct sections of the training data, in addition to being assessed on a wholly autonomous data set to measure its generalization capability.

Instead of a simple random splitting, a stratified random splitting^61^ was applied to ensure the same proportion of active (1) and inactive (0) compounds across data sets, and the data remained unbalanced toward the inactive class. A test suite was implemented to guarantee no data leakage happened in the data splitting procedure (https://github.com/carineribeirost/PI3K-split-evaluation).

RF hyperparameters were tuned by a stratified nested *k*-fold cross-validation (CV)^62^ procedure using a series of train/validation/test set splits with *GridSearchCV* (exhaustive combination of RF parameters) in the inner 5-fold CV loop along with *cross_val_score* in the outer 10-fold CV loop to evaluate individual model performance. The resulting scores with the best parameters were used to select 10 models, and a consensus model was built by averaging the outputs of these models.

### Model Performance Evaluation and Interpretation

The consensus model was evaluated against the external set using a 5-fold CV procedure. Model performance was assessed by the following scores: balanced accuracy (BACC) (eq 2), sensitivity (SE) (eq 3), specificity (SP) (eq 4), Matthew’s correlation coefficient (MCC) (eq 5), positive predictive value (PPV) (eq 6), and negative predictive value (NPV) (eq 7), which are based on the four components of a confusion matrix, i.e., true positives (TP), true negatives (TN), false positives (FP), and false negatives (FN).

BACC reflects a classifier’s ability to correctly predict both positive and negative outcomes, accounting for class imbalance by averaging SE and SP. SE and SP are utilized to demonstrate the classifier’s proficiency in accurately categorizing positive and negative instances. The MCC^64^ offers a well-balanced assessment, derived from the confusion matrix, which reflects the distribution of TP, TN, FP, and FN. This metric delivers a comprehensive appraisal of the model’s predictive performance by considering all aspects of the confusion matrix, thus furnishing an impartial measure, where MCC = +1 for perfect classification, MCC = 0 for random classification, and MCC = −1 for perfectly wrong classification. Additionally, PPV and NPV indicate the proportions of correctly identified positive and negative outcomes as TP and TN, respectively, showcasing the practical precision of the classifier.

2 $$\mathrm{BACC}=\frac{1}{2}\left(\frac{\mathrm{TP}}{\mathrm{TP}+\mathrm{FN}}+\frac{\mathrm{TN}}{\mathrm{TN}+\mathrm{FP}}\right)$$

3 $$\mathrm{SE}=\frac{\mathrm{TP}}{\mathrm{TP}+\mathrm{FN}}$$

4 $$\mathrm{SP}=\frac{\mathrm{TN}}{\mathrm{TN}+\mathrm{FP}}$$

5 $$\mathrm{MCC}=\frac{\mathrm{TP}\times \mathrm{TN}-\mathrm{FP}\times \mathrm{FN}}{\sqrt{\left(\mathrm{TP}+\mathrm{FP}\right)\times \left(\mathrm{TP}+\mathrm{FN}\right)\times \left(\mathrm{TN}+\mathrm{FP}\right)\times \left(\mathrm{TN}+\mathrm{FN}\right)}}$$

6 $$\mathrm{PPV}=\frac{\mathrm{TP}}{\mathrm{TP}+\mathrm{FP}}$$

7 $$\mathrm{NPV}=\frac{\mathrm{TN}}{\mathrm{TN}+\mathrm{FN}}$$

The precision–recall curve offers an alternative method for evaluating model performance by plotting precision (PPV) against recall (SE) across different classification thresholds.^65^ The area under the curve of the precision recall (AUC-PR) provides a summary of classifier performance, focusing on the positive class.

A higher AUC-PR indicates a better balance between precision and recall, reflecting the model’s ability to identify true positives while minimizing false positives. This metric is particularly valuable for imbalanced data sets, where traditional metrics, such as receiver operating characteristic (ROC), may yield misleading results by considering both classes. AUC-PR is thus more informative in evaluating classifiers in scenarios where the positive class is of primary interest.^65^

For RF model interpretation, the feature importance of Morgan fingerprints was determined using the SHapley Additive exPlanations (SHAP) method^66^ implemented in the SHAP Python package (v.0.45.1).

A final ensemble model was trained following the same procedure as before (10 models selected following hyperparameter tuning in a nested CV) using the original data set (combination of internal set and external set) after the model quality was assessed.

### Applicability Domain Analysis and Model Application

The subset of FDA-approved drugs available in the DrugBank database^67,68^ (https://go.drugbank.com/) was used as an application set to search for a possible PI3K pathway inhibitor using our ensemble model.

The application set was standardized following the same procedure as the original set, and its canonized SMILES data were used to calculate Morgan fingerprints; the same features of the training set were selected.

In order to assess the applicability domain^69^ of the RF ensemble model in the application set, two distance-based methods were employed: *k*-nearest neighbors (*k*-NN) and leverage.^70^ The *k*-NN technique evaluates the likeness of data points based on their feature vectors, using Mahalanobis distance to gauge proximity in the feature space.^71^ It recognizes the *k*-nearest neighbors of a query point and utilizes them for forecasting or categorization purposes.^71^ It was calculated with the scikit-learn nearest neighbors method (https://scikit-learn.org/stable/modules/neighbors.html).

The leverage of a query chemical correlates with its Mahalanobis distance from the training set centroid. Calculated using the leverage matrix, diagonal values indicate leverage for each data point, with higher values suggesting a greater influence on the model. Leverage can signal unreliable predictions when exceeding a warning threshold, typically three times the average leverage, indicating that the query lies outside the descriptor space.^70^ The leverage for the data set was calculated with eq 8.

8 $$H=X{\left(X^{T}X\right)}^{-1}X^{T}$$

In eq 8, *X* is the matrix of molecular descriptors and *H* is the leverage matrix, and the diagonal elements of *H* represent the leverage values for each compound.

### Correlation of Predicted Bioactivity with Binding Affinity (Molecular Modeling, Docking, and Dynamics Simulations)

#### Structure Preparation and Validation

In order to create a feasible human PI3Kα protein structure model for molecular docking and dynamics simulations, the X-ray diffraction crystal structure of the PI3Kα, which contains p110α and p85α subunits in a complex with alpelisib (a PI3Kα inhibitor that binds to the ATP pocket in the p110α kinase domain), was obtained from the Protein Data Bank (PDB) (https://www.rcsb.org/).

There are eight available human PI3Kα complexes for alpelisib (PDB ID: 4JPS, 7MYO, 7PG6, 8GUA, 8GUB, 8GUD, 8V8U, and 8V8V). 4JPS has the highest value for resolution among the complexes without mutation (resolution: 2.20 Å). Some regions of amino acid residues relating to subunits p110α (i.e., 1–1, 228–243, 314–323, 498–524, 864–871, and 1062–1068) (UniProtKB: P42336, PK3CA_HUMAN) and p85α (i.e., 301–331, 364–366, 387–390, 399–422, 431–433, and 593–593) (UniProtKB: P27986, P85A_HUMAN) are missing from this crystal structure (4JPS).

Therefore, comparative/homology modeling was performed to fill the missing regions in both subunits by employing ModWeb (https://modbase.compbio.ucsf.edu/modweb/),^72^ a web server that relies on the MODELLER (https://salilab.org/modeller/) software^73^ for automated comparative modeling. The server selects templates based on E-values, generates multiple models, and identifies the best model using DOPE statistics. Model evaluation was performed using the GA341, Z-DOPE, MPQS, and TSVMod NO35 (predicted native overlap 3.5 Å) scores and TSVMod RMSD (predicted RMSD).^72,74–76^

Thus, the following UniProtKB sequences were used as targets: P42336 (PK3CA_HUMAN) for p110α and P27986 (P85A_HUMAN) for p85α, while the following 3D structures from PDB were used as templates: 5DXH (pdb_00005dxh; resolution: 3.00 Å; A chain, *Homo sapiens* p110α; B chain, *Bos taurus* p85α), 4YKN (pdb_00004ykn; resolution: 2.90 Å; A chain, *Homo sapiens* p110α), and 4JPS (pdb_00004jps; resolution: 2.20 Å; *Homo sapiens*, A chain, p110α, and B chain, p85α).

The model was further refined through molecular dynamics simulations for 10 ns using the GROMACS (v.2022 or v.5.0)^77^ package, applying the CHARMM36 force field^78^ following the protocol for system preparation described previously by our research group.^34^ The protein model was included in a periodic triclinic box (dimensions: 15.960 × 14.051 × 10.356 nm and box volume: 2322.37 nm^3^), solvated with the TIP3P model of water, and neutralized with two Cl^–^ ion atoms. The quality of the model structure was assessed by ERRAT,^79^ Verify3D,^80,81^ and PROCHECK^82,83^ on the SAVES (v.6.0) web server (https://saves.mbi.ucla.edu/).

#### Molecular Docking

The 3D coordinates of the FDA-approved drugs selected from the DrugBank database within the RF model were extracted from the PubChem database (https://pubchem.ncbi.nlm.nih.gov/).^84^ The molecular docking was performed using GOLD (Genetic Optimization for Ligand Docking) (v.2022.3) software.

The docking protocol was validated by redocking, removing the bound inhibitor (alpelisib) from the complex, docking it at the human PI3Kα ATP binding site, and testing the following scoring (or fitness) functions: Piecewise Linear Potential (ChemPLP^85^), GoldScore,^86^ ChemScore,^87^ and Astex Statistical Potential (ASP^88^), and calculating the root-mean-square deviation (RMSD) between the docked and native pose.

The PI3Kα ATP site docking region was defined as the ATP pocket, located at the p110α kinase domain where the reference PI3Kα inhibitor (alpelisib) is bound (PDB ID: 4JPS), which was centered on the Cartesian coordinates (*x* = −2.560000 Å, *y* = −13.722000 Å, *z* = 11.586000 Å) at the alpha-carbon (Cα) of the Val851 residue and included all atoms that lie within the radius (*r*) of the ATP site that was set to 15 Å from the center. The analysis of intermolecular interactions was carried out using BIOVIA Discovery Studio Visualizer (v.2022) software.^89^ Figures were constructed using Visual Molecular Dynamics (VMD) (v.1.9.4) software.^90^

#### Molecular Dynamics

The catalytic activity of the p110α isoform is tightly regulated by an autoinhibition mechanism mediated by the p85α regulatory subunit. Specifically, the N-terminal SH2 (nSH2) domain of p85α directly interacts with the catalytic subunit, locking the enzyme in a basally inactive state. This inhibited conformation is defined by distinct structural features, including a “collapsed” activation loop (a-loop) and an “IN” orientation of the kα11 helix within the kinase domain. A key functional consequence of this arrangement is the distance of over 6 Å between the γ-phosphate of ATP and the lipid substrate binding site, which is too great to permit phosphoryl transfer.^91,92^

The transition to a catalytically active state is an allosteric event triggered by the release of the nSH2 domain from p110α. This dissociation initiates a cascade of conformational changes, causing the a-loop to become “extended” and the kα11 helix to reorient to an “OUT” conformation. This structural reorganization is crucial, as it reduces the distance between ATP and the substrate to a catalytically competent range of approximately 2–3 Å, thereby enabling the phosphorylation of PIP2. The oncogenic mutations frequently observed in the *PIK3CA* gene often function by mimicking these dynamic events, thereby bypassing physiological inhibition and stabilizing the enzyme’s active conformation.^91,92^

Molecular dynamics simulations were carried out using the GROMACS (v.2022 or v.5.0) package,^77^ applying the CHARMM36 force field^78^ following the protocol for system preparation described previously by our research group.^34^

The protein–ligand complexes from docking poses were included in a periodic triclinic box (dimensions: 15.960 × 14.051 × 10.356 nm and box volume: 2322.37 nm^3^), solvated with the TIP3P water model, and neutralized with two Cl^–^ ion atoms.

The compressed trajectory from the protein was centered in Chain A. RMSD, RMSF, hydrogen bonds (H-bond), pairwise interatomic distances (non-H-bond), solvent-accessible surface area, and cluster analysis (cutoff of 0.4 nm) were performed using the *gmx rms*, *gmx rmsf*, *gmx hbond*, *gmx distance*, *gmx sasa*, and *gmx cluster* modules, respectively, available in the GROMACS package.^93^ H-bond frequencies were calculated with HbMap2Grace software (https://github.com/LMDM/hbmap2grace/tree/main),^94^ considering a cutoff of 0.4 nm.

The binding free energy (Δ*G*_bind_) was calculated by the molecular mechanics/Poisson–Boltzmann surface area (MM/PBSA) method^95^ considering the frames identified by cluster analysis (cutoff = 0.10 nm), applying the *g_mmpbsa* package (v.5.1.238) (https://github.com/RashmiKumari/g_mmpbsa).^95^ The energy contribution of residues was calculated using the *MmPbSaDecomp.py* and *MmPbSaStat.py* scripts.^95^ Figures of the interactions and trajectory analysis were composed using VMD (v.1.9.4)^90^ and PyMOL (v.3.8.5)^96^ softwares.

## Results and Discussion

### Model Choice

Among the evaluated models, the *random forest classifier* was chosen for this study, as it demonstrated the best overall performance across several metrics (accuracy, balanced accuracy, ROC AUC, and F1-score), closely followed by other tree-based ensemble algorithms such as *ExtraTreesClassifier* and *XGBClassifier* (Figure S1).

Although the *KNeighborsClassifier* achieved comparable accuracy, it was not chosen as the primary predictive model due to its limited scalability and reduced generalization capacity in high-dimensional chemical feature spaces.^97^ Distance-based models such as *k*-nearest neighbors typically perform poorly with large and sparse molecular fingerprint representations, while ensemble tree methods such as random forests provide a more robust balance between predictive power, interpretability, and resilience to overfitting.

### Selection of Optimal Parameters and Model Development

A MODI of 0.909 for Morgan fingerprints indicates that the data set is reliable for classification modeling. Post elimination of one pair of intercorrelated features, the Morgan fingerprint descriptor count was refined from 2048 to 31. The bias toward the inactive class is manageable through an assessment of the adequacy of sensitivity and a high positive predictive value (PPV),^98^ since these metrics ensure that the model is capable of correctly identifying active instances, which is crucial for the model.

Figure 2 provides a comprehensive analysis of the chemical structures of these prominent features. Morgan fingerprints utilize fixed substructures specified by the fingerprint length and radius. In the substructure illustrations shown in Figure 2, blue indicates the central atom, yellow highlights the aromatic atoms, and dark gray emphasizes the aliphatic ring atoms. Additionally, light gray is used to represent atom/bond structures that affect the atom’s connectivity invariants but are not directly included in the fingerprint.

**Figure 2:**
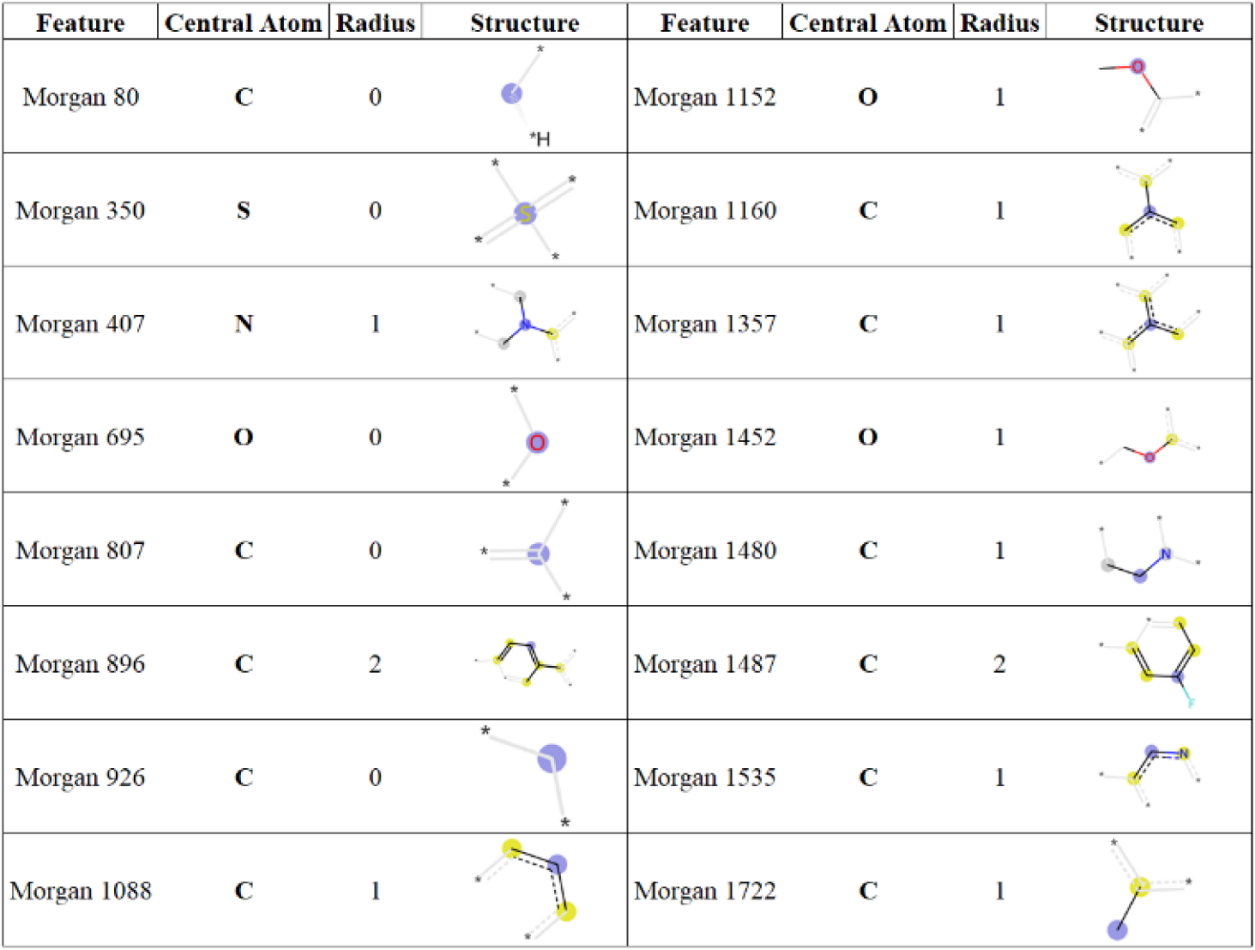
Selected Morgan fingerprint chemical structures.

To construct a prediction model of PI3Kα inhibition, 31 features from Morgan fingerprints were used as inputs for the model’s construction and 50 models were evaluated through nested cross-validation using RF as the machine learning method. An ensemble model was further derived by averaging the predictions of the ten best models. Table 1 lists the individual performances of the 10 best models as well as the consensus model performance, with both cases evaluated against the external set.

**Table 1:** Performances of the Ten Best Models and the Ensemble Model on the External Set^a^

Model #	AUC	BACC	SE	SP	MCC	PPV	NPV
**Model 1**	0.8839	0.8763	0.8430	0.9307	0.7764	0.8604	0.9203
**Model 2**	0.8980	0.8745	0.8321	0.9326	0.7761	0.8631	0.9188
**Model 3**	0.9022	0.8700	0.8212	0.9288	0.7827	0.8587	0.9254
**Model 4**	0.8964	0.8764	0.8285	0.9382	0.7517	0.8675	0.9011
**Model 5**	0.8946	0.8690	0.8285	0.9325	0.7761	0.8631	0.9188
**Model 6**	0.9016	0.8764	0.8285	0.9232	0.7750	0.8493	0.9249
**Model 7**	0.8965	0.8791	0.8394	0.9176	0.7522	0.8370	0.9159
**Model 8**	0.8942	0.8782	0.8358	0.9270	0.7473	0.8494	0.9066
**Model 9**	0.8939	0.8773	0.8285	0.9382	0.7811	0.8726	0.9176
**Model 10**	0.8919	0.8801	0.8266	0.9195	0.7488	0.8389	0.9126
**Ensemble**	**0.8980**	**0.8981**	**0.8376**	**0.9288**	**0.7708**	**0.8566**	**0.9185**

a AUC, recall–precision area under the curve; BACC, balanced accuracy; SE, sensitivity; SP, specificity; MCC, Matthew’s correlation coefficient; PPV, positive predictive value; NPV, negative predictive value.

Accuracy (90%) and recall–precision AUC (96%) (Figure 3) showcase the model’s ability to make reliable predictions. Furthermore, specificity (93%) suggests that potential active compounds are less prone to being overlooked, thus reducing the occurrence of false negatives. Sensitivity (84%) and PPV (86%) imply that while the model may identify fewer active compounds, those identified are highly probable to be true positives.

**Figure 3:**
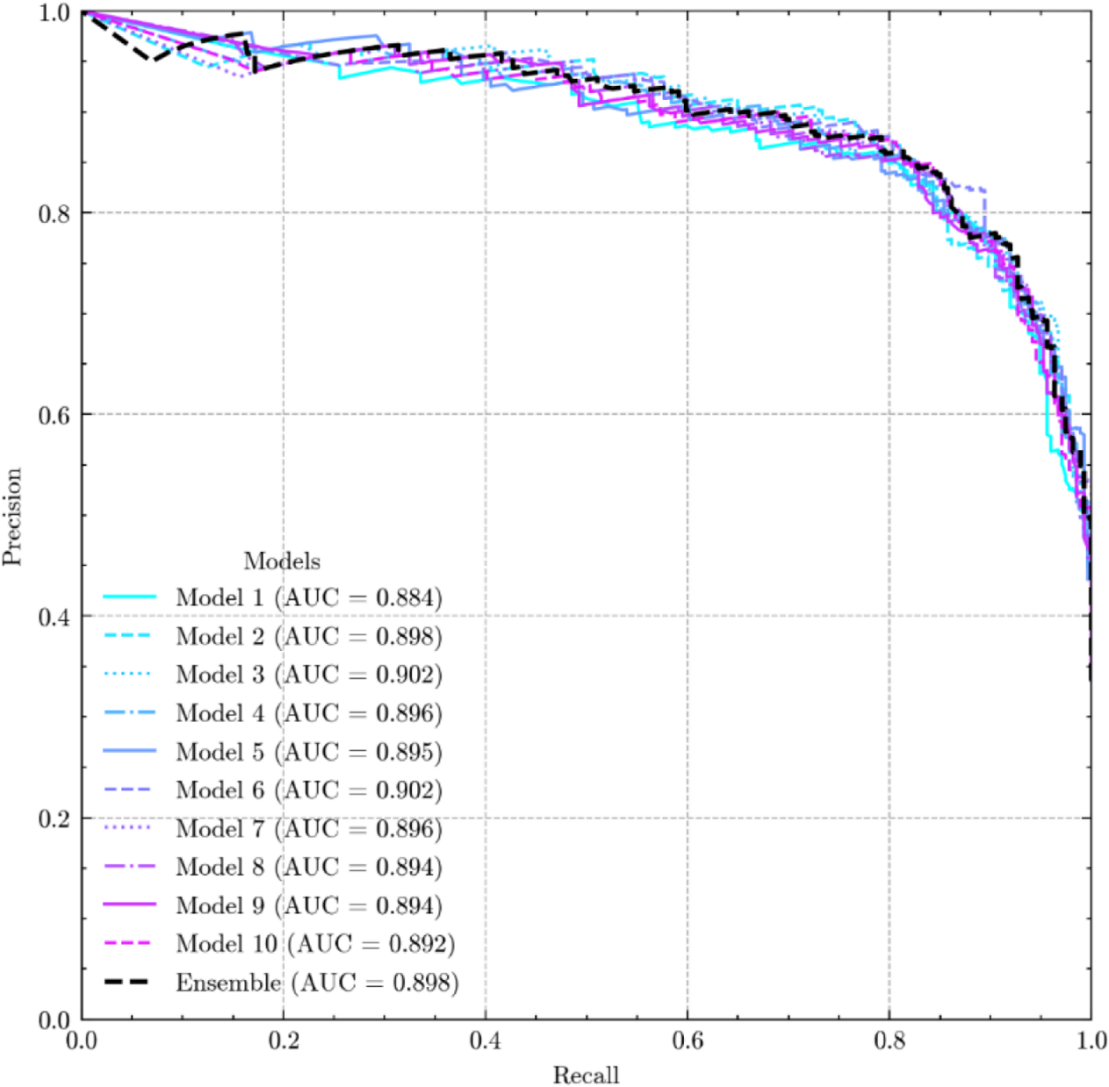
AUCs of the ten RF best models on the external set.

### Model Explainability

In this study, we used the SHAP algorithm to calculate the impact of each Morgan bit (feature) on the model output. Shapley is a method that assigns values to the contribution of each variable to the model’s outcome.^66^

One way to visualize these results is through a beeswarm plot. This plot includes three dimensions: the *y*-axis lists all model features in order of importance, the *x*-axis shows the SHAP values (positive or negative contributions), and the color represents the feature’s value. The beeswarm plot avoids overlapping data points, allowing the density of the results to be clearly observed.

Our model features a binary variable (i.e., biological activity, “*y*” dependent variable, active or inactive). Points on the left side of the *x*-axis indicate inactive outcomes, while points on the right indicate active outcomes. Blue color signifies that the feature is absent in the molecule, while red indicates its presence.

In summary, red points on the right indicate that the feature’s presence is associated with molecule activity, while blue points on the left correspond to inactivity. Conversely, red points on the left and blue on the right suggest an opposite relationship (Figure 4).

**Figure 4:**
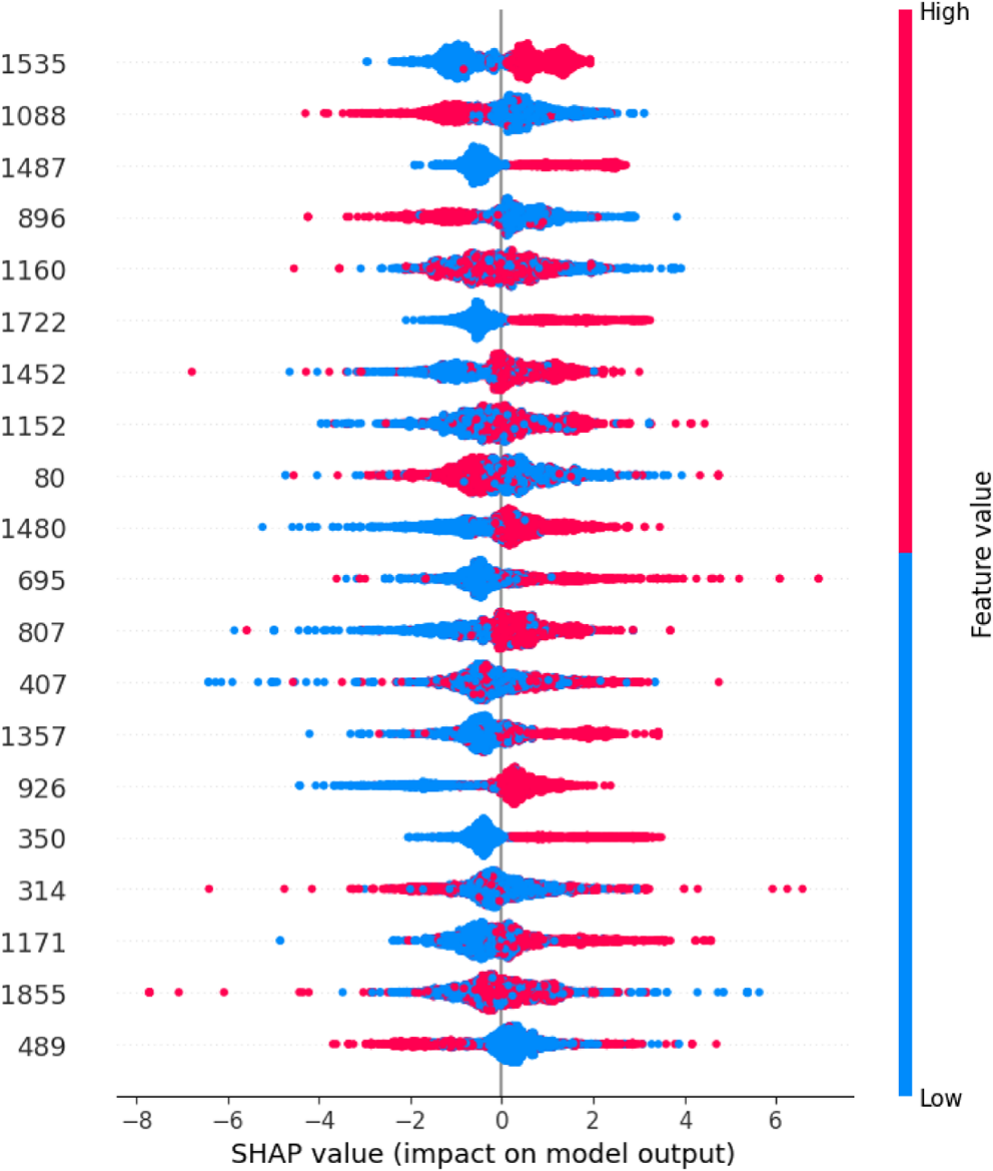
SHAP dependence plot of the first 20 Morgan fingerprint features for the ensemble model. SHAP values were normalized by the mean and standard deviation.

We can analyze the impact of each Morgan feature bit in the model individually in the beeswarm plot (Figure 3). A few illustrative cases are Morgan bits 350, 1535, 875, 1452, and 1160 (Figure 2). Feature 350 (central atom = S, and radius = 0) exhibits a dense cluster of blue points on the left side of the axis, indicating that molecules lacking this feature tend to be inactive. Feature 1535 shows two clear clusters: blue points on the left and red points on the right. Its presence indicates activity, while its absence corresponds to inactivity. Feature 875 displays a contrasting pattern with a high concentration of red points on the left side of the axis. Feature 1452 shows a less distinct distribution: blue points appear on both sides, and red points are scattered across the axis. Overall, the presence of this feature tends to have a positive influence on activity, whereas its absence appears to have a negative effect. Feature 1160 presents a mixed pattern with blue and red points interspersed. Because the beeswarm plot does not display overlapping points, it is difficult to clearly determine whether this feature’s presence promotes or reduces activity. Morgan bits that display only centralized blue clusters likely have limited interpretability, as these features appear infrequently across the data set.

### Applicability Domain

After assessing the thresholds set by *k*-NN (7.6060) and leverage (0.0228) for the entire PI3Kα ChEMBL set and the application set (i.e., DrugBank/FDA-approved drugs data set), no outlier compounds were observed. This indicates that the chemical space exhibits a strong similarity between both data sets (Figure 5).

**Figure 5:**
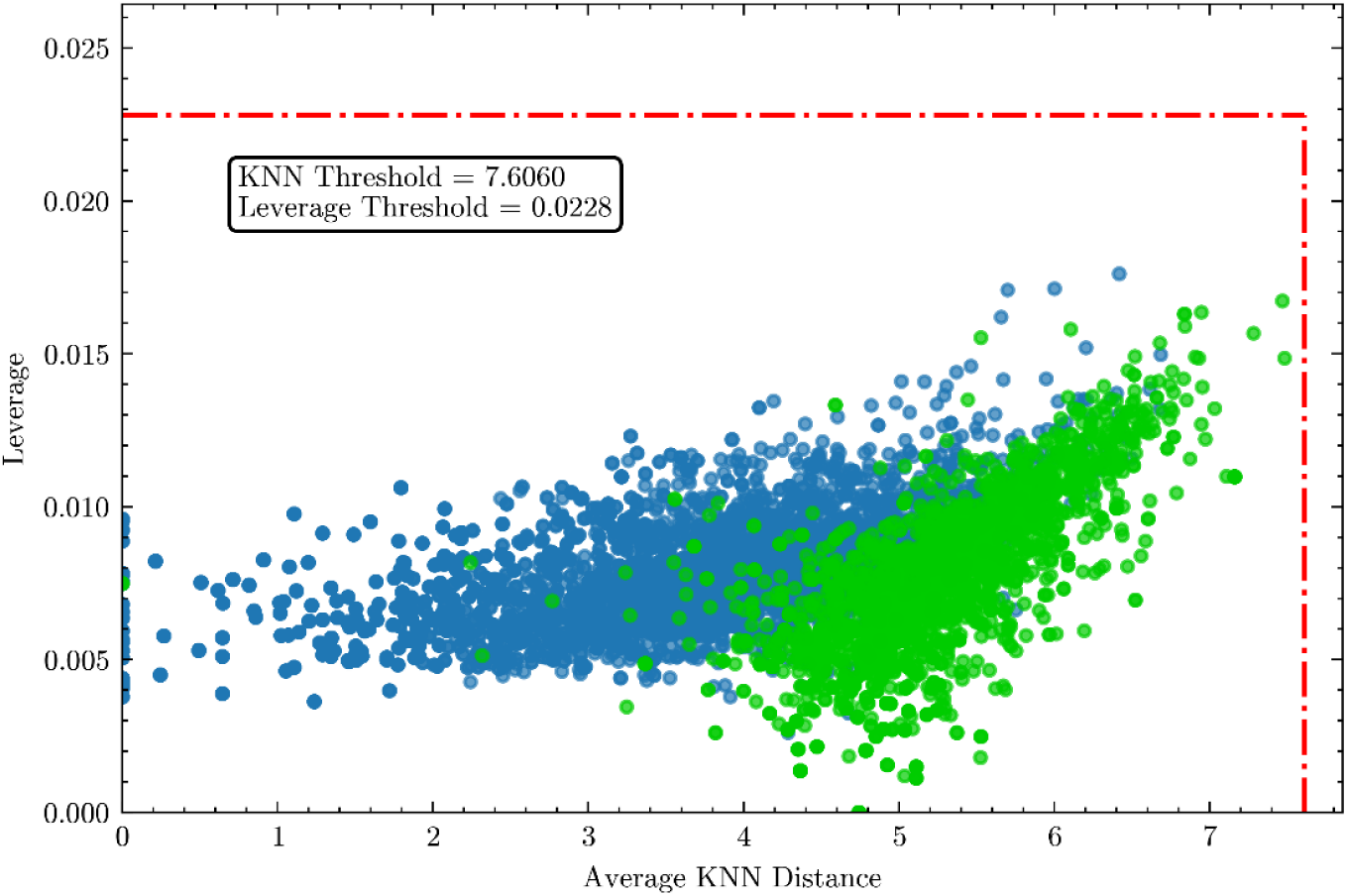
Analysis of the ChEMBL PI3Kα inhibitors and DrugBank/FDA-approved drugs data set within the applicability domain using *k*-nearest neighbors (*k*-NN) with Euclidean distance and leverage. Legend: blue dots (internal data set), green dots (external data set).

### Model Application on the DrugBank/FDA-Approved Drugs Data Set

We employed the PI3Kα random forest (RF) model to predict the activity of 2607 FDA-approved drugs from the DrugBank database. For subsequent analysis, we selected five compounds that exhibited a predicted probability of activity of >60% against the human PI3Kα target. These compounds, detailed in Table 2, are ceritinib (**CER**), fursultiamine (**FUR**), simeprevir (**SIM**), trofinetide (**TRO**), and vemurafenib (**VEM**).

**Table 2:**
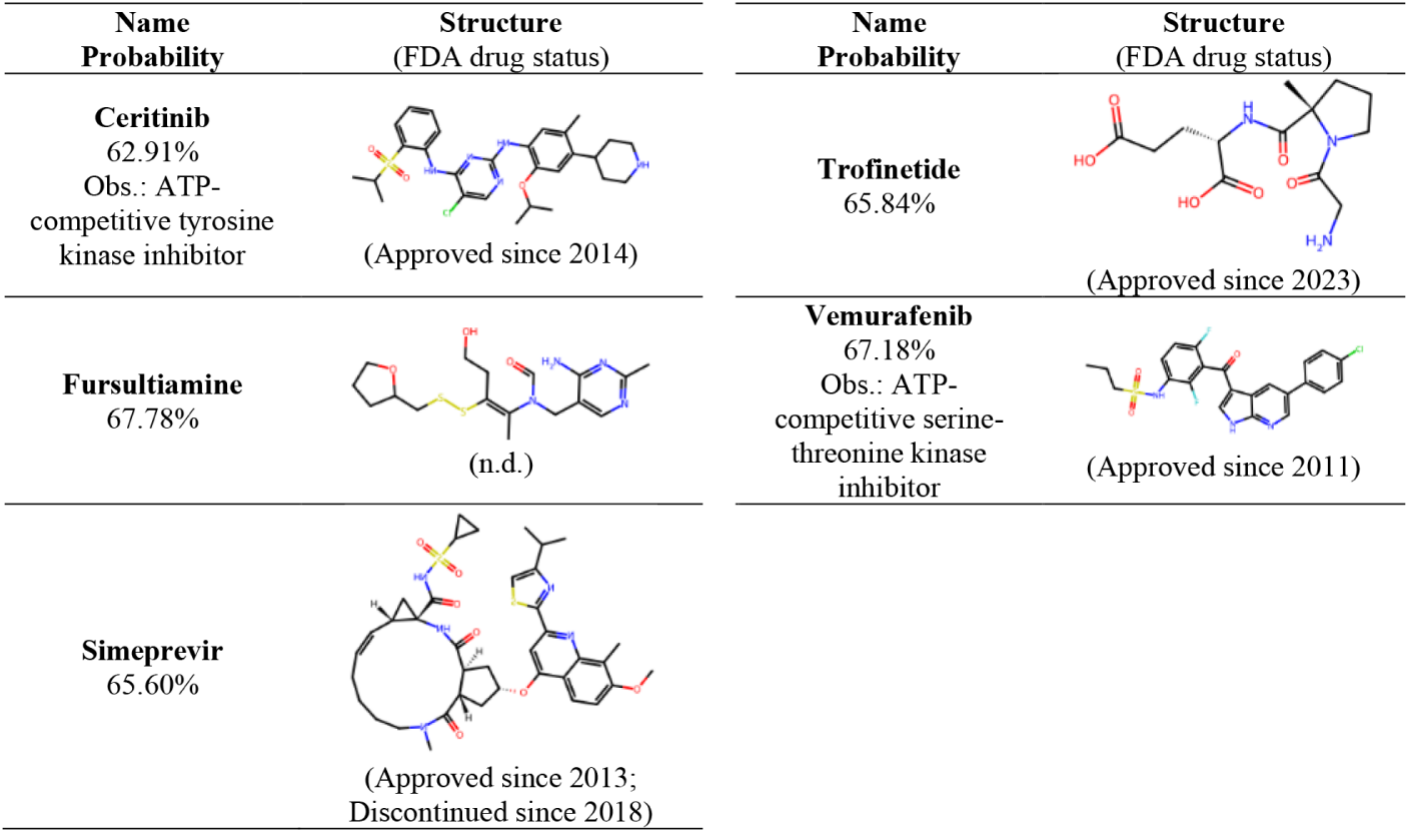
Top Five DrugBank/FDA-Approved Drugs with More Than 60% Probability of Being Active for the Human PI3Kα Target According to the RF Model (FDA Drug Status from the FDA-Approved Drugs Site)^a^

a https://www.fda.gov/drugsatfda

In our analysis of the compounds predicted as active, we first observed a set of recurring structural features, and we found that fingerprints 1152 (**CER**, **SIM**, **TRO**, **VEM**), 807 (**FUR**, **SIM**, **TRO**, **VEM**), and 926 (**CER**, **FUR**, **SIM**, **TRO**) are present in each of the four molecules. An examination of the SHAP plot for these features indicates that the model generally interprets their presence as a positive contributor to the activity score.

In the case of ceritinib, for instance, we attribute its score to the presence of two high-ranking SHAP features (1535 and 1088). For vemurafenib, we noted the presence of the positive influential bit 1535. However, we also found that it is the only compound in the group to feature bit 1487, a fingerprint we observe to be associated with inactivity. Simeprevir presents a different profile; our analysis shows that it shares more fingerprints with the reference compound than any other molecule.

### Homology Modeling, Structural Validation, and Molecular Docking

Each PI3Kα subunit (i.e., p110α and p85α) was modeled individually, using the PDB structures 5DXH, 4YKN, and 4JPS as templates. The highest values for the GA341, MPQS, and TSVMod NO35 scores, along with the lowest TSVMod RMSD and the most negative Z-DOPE score, were used as combined criteria to select the best model structures generated by ModWeb. The minimum Z-DOPE score calculated for p110α was −1.07766, whereas for p85α, it was −1.20526. The calculated MPQS for p110α was 2.1066 and its TSVMod NO35 score was 0.854, whereas for p85α, these scores were 1.56852 and 1.0, respectively; both models achieved an ideal GA341 score of 1.0 and a TSVMod RMSD below 2.0 Å.

Subsequently, these two subunits were assembled with PyMOL (v.3.8.5)^96^ to form the overall PI3Kα heterodimer structure. After conducting a 10 ns molecular dynamics simulation (MDS), the most representative cluster from the trajectory was selected and analyzed by ERRAT,^79^ Verify3D,^80,81^ and PROCHECK^82,83^ on the SAVES (v.6.0) web server (https://saves.mbi.ucla.edu/). The validation results for the 3D model refined by MDS and the comparison with the experimental 3D structures (PDB ID: 4JPS, 4YKN, and 5DXH) used as templates are available as Supporting Information (Figures S2–S6, Verify 3D plots; Figures S7–S16, ERRAT plots; Figures S17–S23, Ramachandran plots).

ERRAT identifies poorly modeled regions in proteins by analyzing intermolecular interactions and comparing them with high-quality structures. The ERRAT score for the raw model was computed as 75.00, while the score for the optimized model was 94.84 (Table S1, Supporting Information).

Verify3D validated the compatibility between the 3D structure of a protein and its 1D amino acid sequence by comparing it with high-quality structures. 79.80% of the residues averaged a 3D-1D score ≥0.1 for the optimized model, compared to 77.78% for the raw model (Table S1, Supporting Information). The Ramachandran plot was used to evaluate the energetically viable regions of the PI3Kα model with PROCHECK (Figures S17–S23, Supporting Information).

For the raw model, 93.5% of residues lie in the most favored regions, 5.9% in additionally allowed, 0.4% in generously allowed, and 0.2% in disallowed regions. In the final, refined model, 91.2% of residues settled into the most favored regions, with 8.1% in additionally allowed, 0.7% in generously allowed, and 0.0% in disallowed regions. While the percentage in the most favored regions slightly decreased compared to the initial model (93.5%), the refinement successfully eliminated all stereochemically disallowed conformations, indicating a more stable and viable overall structure.

The docking protocol was validated by redocking, where the inhibitor (alpelisib) was removed from the complex (4JPS), and the root-mean-square deviation (RMSD) was calculated between the docked and native poses. The results showed a higher score for ChemPLP (95.1295) (Table 3) compared to GoldScore (76.3859), ASP (50.5148), ChemScore (46.2043), and an RMSD of 0.3123 Å, indicating a close superposition between the docked and native poses.

**Table 3:** Redocking Scores and RMSD (Å) Obtained with GOLD Software

Score Function	Score	RMSD
**ASP**	50.5148	0.3929
**ChemPLP**	95.1295	0.3123
**ChemScore**	46.2043	0.2697
**GoldScore**	76.3859	0.2641

Key interactions were observed, including hydrogen bonds with Val851, Ser854, and Gln859 (Figure 6, Table 3), as well as engagement with the hydrophobic pocket consisting of Arg770, Met772, Ser774, Pro778, Ile800, Lys802, Tyr836, Ile848, Glu849, Val850, Arg852, Asn853, His855, Thr856, Met922, Phe930, Ile932, and Asp933.

**Figure 6:**
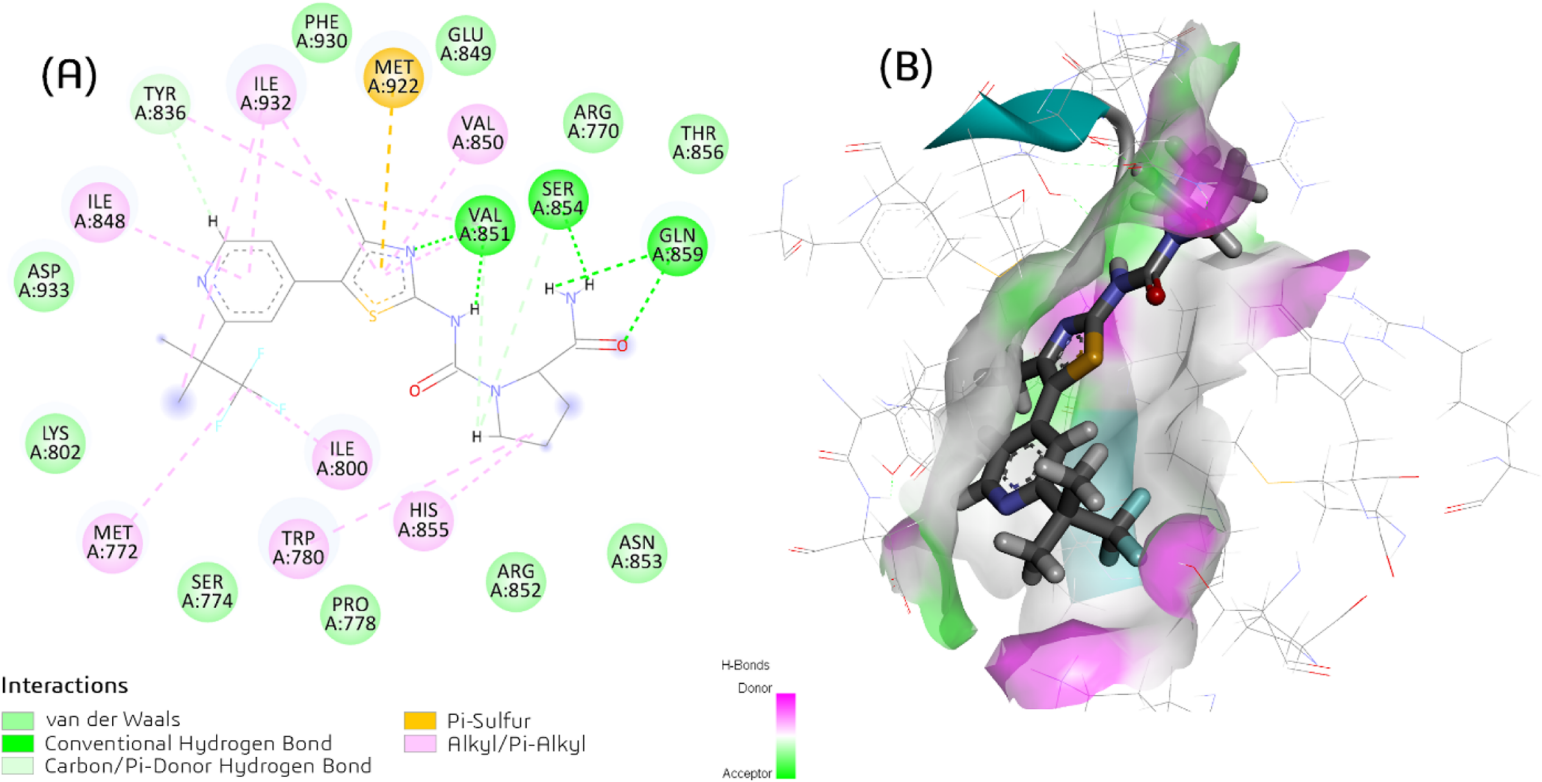
(A) Superposition of the best-scoring docked (GOLD ChemPLP scoring function) and X-ray poses of the alpelisib inhibitor in the ATP binding site of the human PI3Kα model, (B) and the corresponding 2D diagram of the protein–ligand interactions.

Among the potential binding poses predicted by GOLD (ChemPLP) for each protein–ligand system, the one with the highest score was selected as the optimal choice (Table 4). According to ChemPLP scores, the decreasing order of binding was alpelisib (**ALP**; 95.1295) > vemurafenib (**VEM**; 88.9519) > trofinetide (**TRO**; 75.0655) > fursultiamine (**FUR**; 70.6309) > ceritinib (**CER**; 69.2789) > simeprevir (**SIM**; 63.5133) (Table 4).

**Table 4:** Highest ChemPLP Score Binding Poses for the Alpelisib (**ALP**) Inhibitor (Redocking) and the Top Five Compounds (Docking) Predicted as Active (RF Model): Ceritinib (**CER**), Fursultiamine (**FUR**), Simeprevir (**SIM**), Trofinetide (**TRO**), and Vemurafenib (**VEM**)

#	ChemPLP score	H-bond^a^	Residues (H-bond)	vdW^b^	Residues (vdW)
**ALP**	95.1295	4.9440	Val851, Gln859, Ser854	–70.0884	Arg770, Pro778, Lys802, Arg852, Asn853, Glu849, Thr856, Phe930, Asp993
**CER**	69.2789	1.3432	Gln859, Arg770, Arg852	–60.4844	Glu768, Met772, Ile800, Tyr836, Ile848, Glu849, Val850, Asn853, Ser854, Thr856, Phe930
**FUR**	70.6309	5.1079	Val851, Thr856, Ser774	–52.8011	Met772, Ser773, Pro778, Trp780, Ile800, Lys802, Ile848, Val850, Ser854, Gln859, Ser919, Phe930
**SIM**	63.5133	1.3252	Ser774	–60.5765	Ser773, Ala775, Pro778, Lys802, Leu807, Asp810, Ile800, Arg852, Asn853, His855, Thr856, Met858, Gln859, Ser919, Phe930, Asp933
**TRO**	75.0655	1.7740	Tyr836, Val851, Glu849, Asp993, Asp810	–77.4188	Met772, Pro778, Trp780, Leu807, Leu814, Met922, Phe930, Ile932, Phe934,
**VEM**	88.9519	1.9440	Tyr836, Val851, Gln859, Asp933	–79.5912	Met772, Trp780, Glu798, Ile800, Lys802, Asp810, Leu814, Lys838, Asn853, Ser854, Thr856, Phe930, Phe934

a H-bond, hydrogen bond interaction energy (a.u.).

b vdW, van der Waals interaction energy (a.u.).

Hydrogen bond (H-bond) and van der Waals (vdW) interactions between PI3Kα residues and the top six drugs predicted to be active by the RF model were also identified (Table 4). H-bonds were formed with residues Val851, Gln859, Tyr836, and Asp933 for **VEM**; Asp810, Glu849, Val851, Asp993, and Tyr836 for **TRO**; Arg852, Gln859, and Arg770 for **CER**; Ser774 for **SIM**; and Val851, Thr856, and Ser774 for **FUR**; while no H-bonds were observed for **MAR** (Table 4).

To better comprehend how these interactions may vary during simulated motion, the top scored pose of each system was submitted to molecular dynamics simulations.

### Molecular Dynamics Simulations

We conducted a 200 ns molecular dynamics simulation (MDS) of the six protein–ligand aqueous systems, considering as ligands the reference alpelisib (**ALP**) inhibitor and the five compounds under study, i.e., ceritinib (**CER**), fursultiamine (**FUR**), simeprevir (**SIM**), trofinetide (**TRO**), and vemurafenib (**VEM**), to assess whether the binding mode of these compounds in the aqueous dynamic system is similar to that observed with those provided by molecular docking.

The trajectories’ root-mean-square deviation (RMSD) was used to demonstrate the stability of each protein–ligand complex based on protein backbone Cα-atoms (Figure 7A–F) and ligand atom (Figure 8A–F) shifts. Except for the RMSD of protein–**VEM** (Figure 7F), which stabilized from 30 ns with RMSD = 2.80 ± 0.63 Å, all other protein–ligand complexes reached their stabilized status in the initial time of MDS (Figure 7A–E). Protein–ligand complexes of **FUR** (RMSD = 3.61 ± 0.45 Å, Figure 7C), **ALP** (RMSD = 3.74 ± 0.45 Å, Figure 7A), and **TRO** (RMSD = 3.78 ± 0.37 Å, Figure 7E) showed similar RMSD profiles, as well as **SIM** (RMSD = 4.48 ± 0.50 Å, Figure 7D) and **CER** (RMSD = 4.25 ± 0.58 Å, Figure 7B).

**Figure 7:**
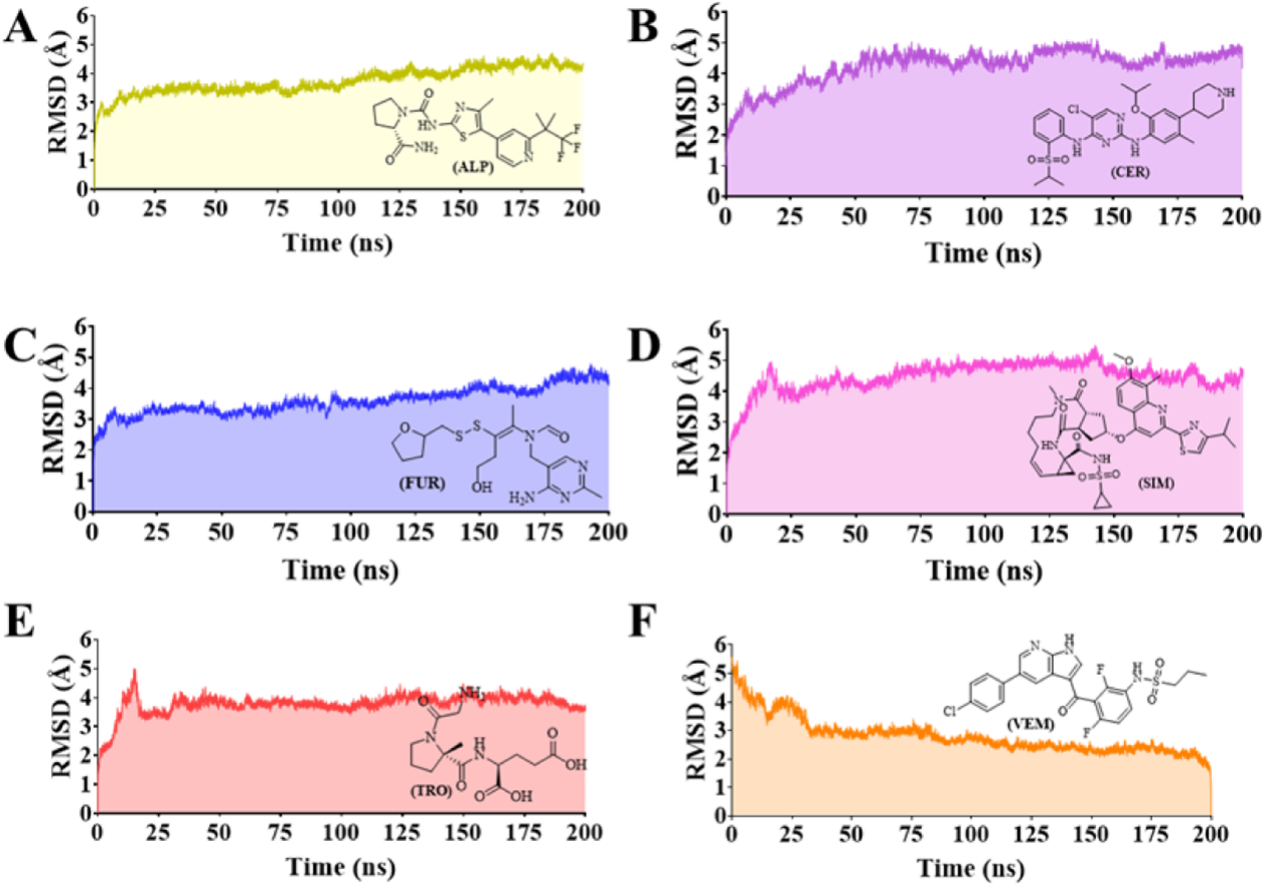
RMSD analysis (Cα-atoms of the protein backbone) from the molecular dynamics simulations of the protein–ligand aqueous systems considering as ligands: (A) **ALP** (alpelisib, reference inhibitor), (B) **CER** (ceritinib), (C) **FUR** (fursultiamine), (D) **SIM** (simeprevir), (E) **TRO** (trofinetide), and (F) **VEM** (vemurafenib).

**Figure 8:**
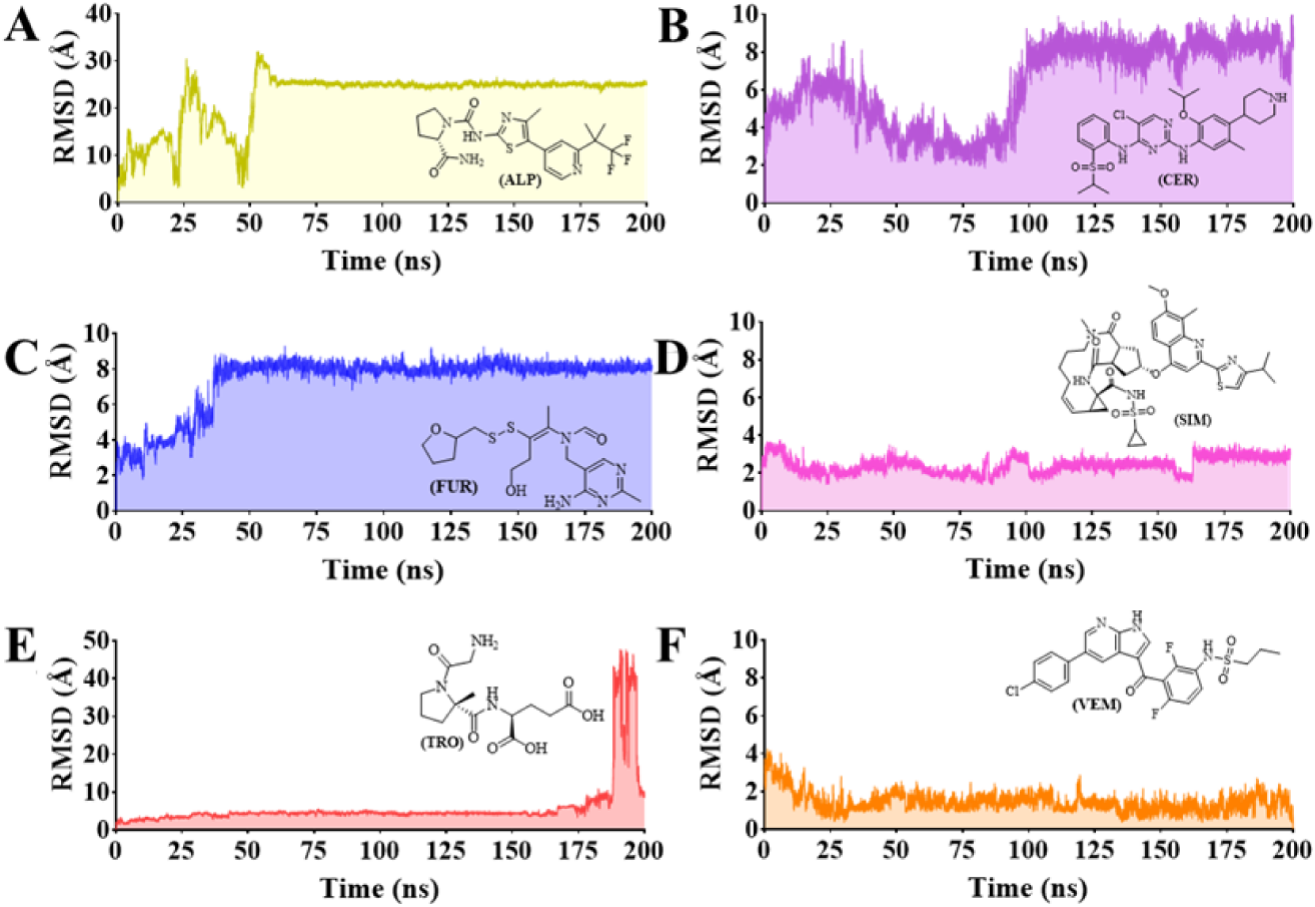
RMSD analysis of the ligand atoms from the molecular dynamics simulations of the protein–ligand aqueous systems considering the ligands: (A) **ALP** (alpelisib, reference inhibitor), (B) **CER** (ceritinib), (C) **FUR** (fursultiamine), (D) **SIM** (simeprevir), (E) **TRO** (trofinetide), and (F) **VEM** (vemurafenib).

In general, the RMSD analysis of the ligand atoms (Figure 8A–F) of the six protein–ligand complexes suggests that **VEM** (RMSD = 1.44 ± 0.53 Å, Figure 8F) and **SIM** (RMSD = 2.43 ± 0.43 Å, Figure 8D) exhibit greater similarity with their corresponding docking poses. These compounds, all containing in their structures a sulfonamide (**SIM** and **VEM**) group as a substituent, displayed RMSD values ranging from ∼1 to ∼4 Å with low standard deviation values. Conversely, **TRO** (RMSD = 6.05 ± 7.07 Å, Figure 8E), **CER** (RMSD = 6.35 ± 2.16 Å, Figure 8B), and **FUR** (RMSD = 7.35 ± 1.62 Å, Figure 8C) demonstrated higher RMSD values compared to their corresponding docking poses.

It is worth noting that **TRO** remained stable for approximately 160 ns before disconnecting from the molecular target (Figure 8D), which impacted its RMSD value during the last 40 ns of the simulation. Furthermore, **FUR** and **CER** compounds exhibited fluctuations starting from 30 ns (Figure 8C) and 100 ns (Figure 8B), respectively, reaching an average RMSD of ∼8 Å until the end of the simulations. The reference **ALP** ligand exhibited instability mainly during the first 50 ns of MDS, with an average RMSD of 10 Å, in comparison to its docking pose, and then it became stable until the end of MDS, but with a high RMSD of 22.3 ± 5.73 Å (Figure 8A).

As stated before, PI3Kα is composed of two subunits: catalytic (p110α) and regulatory (p85α), and each subunit is characterized by several domains and motifs. The catalytic p110α subunit is formed by several domains, regions, and motifs, including the adaptor-binding domain (ABD) (1–108), Ras-binding domain (RBD) (191–291), C2 (330–480), helical (525–696), and kinase (697–1068) domains, and the DFG (933–935) motif, while the regulatory p85α subunit is formed by the domains SH3 (1–85), GAP (115–298), nSH2 (322–430), iSH2 (431–600), and cSH2 (617–724).

We identified the residues with the greatest fluctuations in the kinase region and used the 4JPS crystal with the **ALP** drug as a reference for molecular docking. We investigated the distance between each of the six ligands, including the reference **ALP** inhibitor, and the nitrogen atom of Val851 as a representative of the residues of the **ALP** binding pocket, which is composed of residues that have at least one atom within a radius of 5 Å from the centroid of **ALP**: Arg770, Met772, Ser774, Pro778, Trp780, Ile800, Lys802, Tyr836, Ile848, Glu849, Val850, Val851, Arg852, Asn853, Ser854, His855, Thr856, Gln859, Met922, Phe930, Ile932, and Asp933 (Figure 9A).

**Figure 9:**
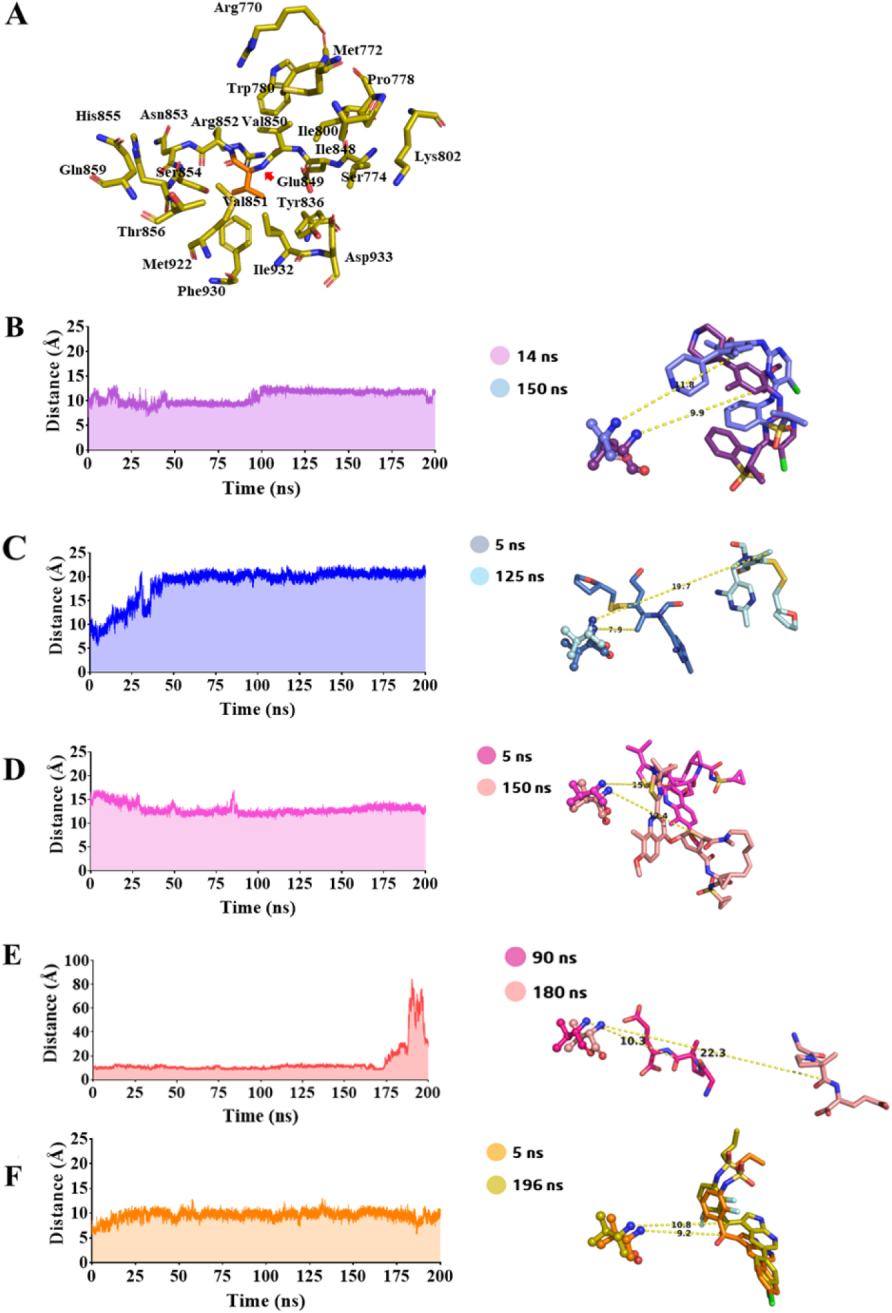
(A) **ALP** binding pocket residues: (PDB ID: 4JPS) Arg770, Met772, Ser774, Pro778, Trp780, Ile800, Lys802, Tyr836, Ile848, Glu849, Val850, Val851 (orange), Arg852, Asn853, Ser854, His855, Thr856, Gln859, and Met922. Average distance (d, Å) analysis (left side) and shortest (*d*_min_) and longest (*d*_max_) distances (right side) of the Val851 N atom and ligands from the 200 ns molecular dynamics simulations of the protein–ligand aqueous systems considering as ligands: (B) **CER** (ceritinib), (C) **FUR** (fursultiamine), (D) **SIM** (simeprevir), (E) **TRO** (trofinetide), and (F) **VEM** (vemurafenib).

Concerning the average distance (*d*) between Val851 and each ligand during the 200 ns of MDS, compounds **VEM** (*d* = 9.53 ± 0.9 Å, Figure 9F) and **CER** (*d* = 10.7 ± 1.3 Å, Figure 9B) had lower and similar profiles, demonstrating low fluctuation around those values during the 200 ns of MDS. **VEM** showed a minimum distance (*d*_min_) of 9.2 Å at 5 ns and a maximum distance (*d*_max_) of 10.8 Å at 196 ns, while **CER** showed a *d*_min_ of 9.9 Å at 14 ns and a *d*_max_ of 11.8 Å at 150 ns (Figure 9F and B). **SIM** (*d* = 13.0 ± 1.1 Å, *d*_min_ = 12.4 Å at 150 ns, and *d*_max_ = 15 Å at 5 ns) and **TRO** (*d* = 14.0 ± 1.2 Å) also assumed poses within similar distances from the Val851 residue evaluated (Figure 9D and E). Except for **TRO**, which has some fluctuation in the final MDS period (*d*_max_ = 22.3 Å at 180 ns, Figure 9E), the other compound (**SIM**) showed stability with low fluctuation from the average distance value. Finally, the greatest average distance of 18.7 ± 3.5 Å was observed for the **FUR** compound with a *d*_min_ of 7.9 Å at 5 ns and a *d*_max_ of 19.7 Å (Figure 9C).

To determine the effect of fluctuations of residues on domains of the p110α subunit, the root-mean-square fluctuations (RMSF) were calculated (Figure 10A–G). All protein–ligand complexes exhibited similar fluctuating behaviors in general, mainly in residues between 851–1062, i.e., from the kinase domain, greater than 4.0 Å (Figure 10H). Also, higher fluctuations of 8 to 10 Å (Figure 10A–G) in residues 320 and 525 from the nSH2 and helical domains were observed. Still, it is important to note that these regions are large loops (Figure 10G), and the fluctuation is expected due to loop modeling, which is a problem in protein structure prediction.

**Figure 10:**
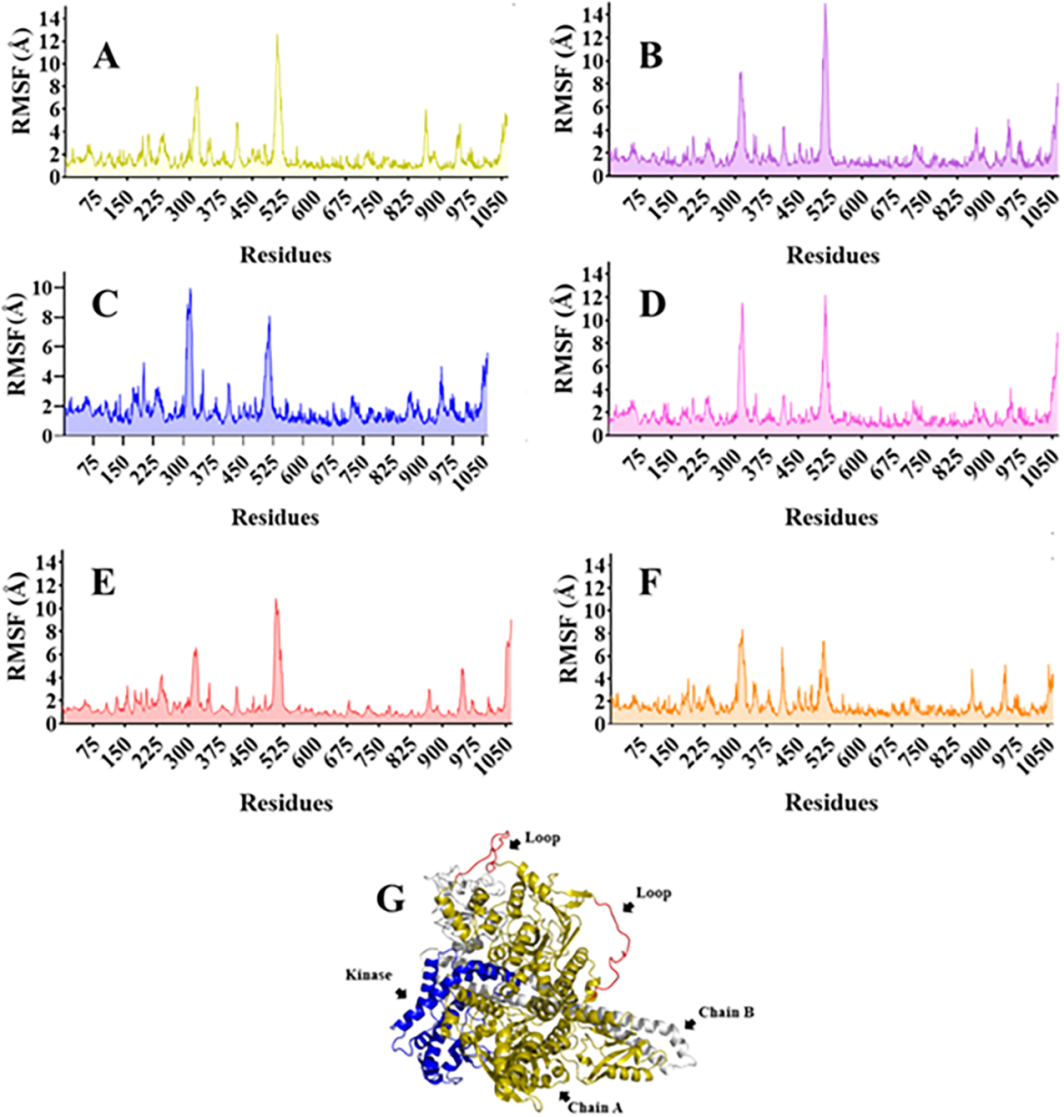
Subunit p110α residues RMSF analysis of 200 ns molecular dynamics simulations of the protein–ligand aqueous systems considering as ligands: (A) **ALP** (alpelisib, reference inhibitor), (B) **CER** (ceritinib), (C) **FUR** (fursultiamine), (D) **SIM** (simeprevir), (E) **TRO** (trofinetide), and (F) **VEM** (vemurafenib). (G) PI3Kα 3D structure highlighting the catalytic kinase domain (blue) of p110α, loops (red), and chains A (p110α) (gold) and B (p85α) (silver).

We have observed intriguing behavior with the **TRO** compound. According to the RMSD analysis, this compound exhibited signs of instability and dissociated from the molecular target after 180 ns of simulation. This behavior was also apparent in our distance analysis. Additionally, the RMSF analysis indicated notable fluctuations for residues 851–1062 of the kinase domain. Upon closer examination of the molecular dynamic trajectory, we observed movement in two protein loops: one consisting of residues Val851–Leu870 (loop-1, Figure 11A), some of them included in a putative binding site from the kinase domain, for which we also investigated the distance profiles, and the other located at the end (C-terminal) of chain A (p110α), specifically Lys1054–Ile1062 (loop-2, Figure 11A). This movement resulted in the creation of an opening that facilitated the expulsion of the ligand from its binding site (Figure 11A). Furthermore, an analysis of the area variation in these loops revealed an increase compared to the initial simulation, the stable phase, and the postligand exit (Figure 11B). We also analyzed the average solvent-accessible surface area (SASA, nm^2^) of residues 770–932, comprising the catalytic site of the PI3Kα kinase domain. We observed a SASA increase of 0.64 Å,^2^ reflected during the same postligand exit period, i.e., 180 ns, and probably due to the movement of the loops (Figure 11C).

**Figure 11:**
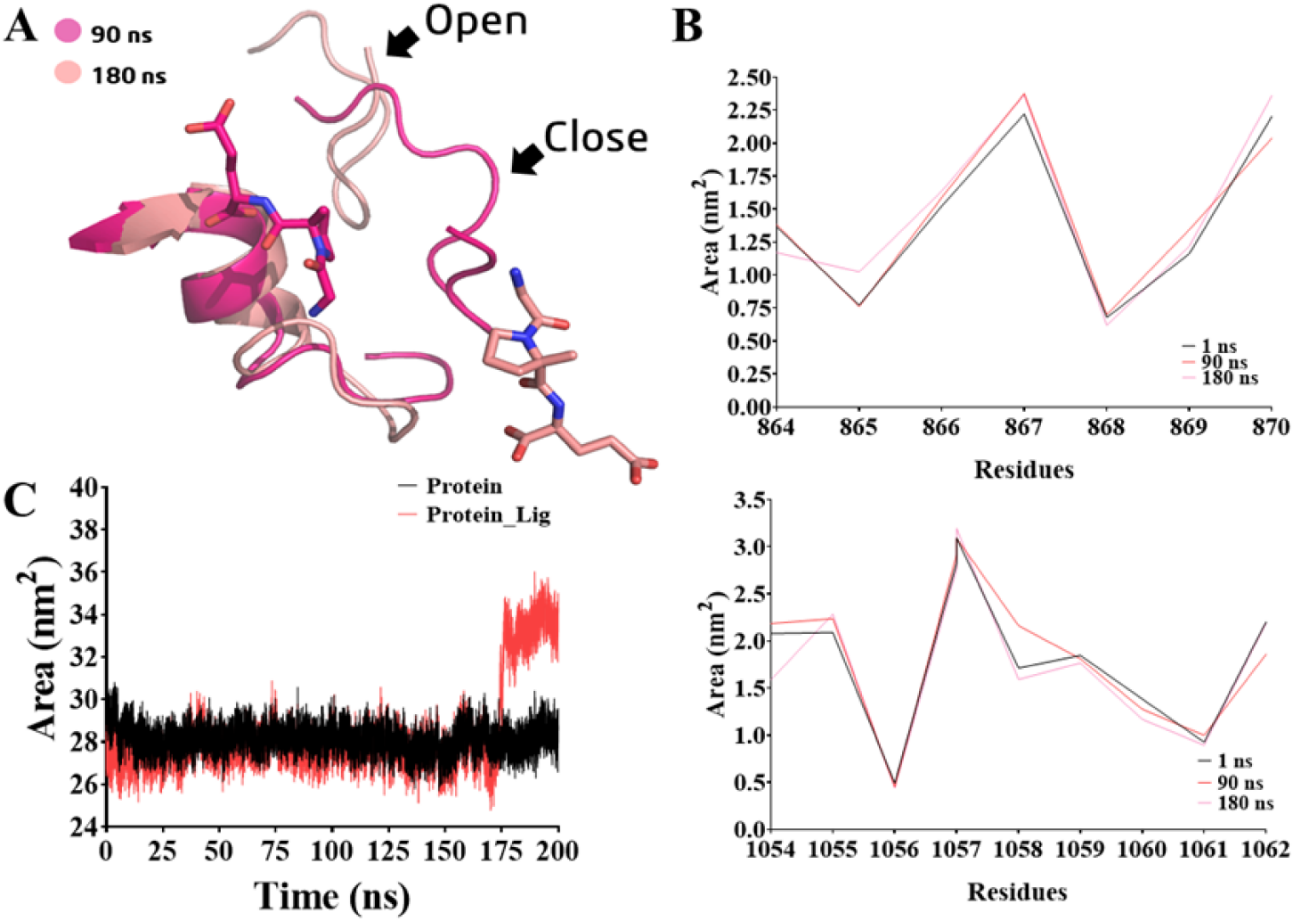
(A) Loop-1 (residues 851–870) and loop-2 (residues 1054–1062) in the presence of trofinetide (**TRO**) in two time periods. (B) Variation in the area (nm^2^) of loop-1 and loop-2 related to the presence of **TRO**. (C) Average solvent-accessible surface area (SASA, nm^2^) of residues 770–932, comprising the PI3Kα catalytic site.

The same profile of loop movement was also observed for **SIM**. We identified that the cyclopropyl sulfonyl substituent influenced the interaction with the residues of the loops, enabling them to open (Figure 12A). There was a slight impact on the surface area (Figure 12A1), and the increase in the SASA value in **SIM** was 8.71 Å^2^ (Figure 12A2). To provide more details about the study, we performed the analysis for all other compounds, but this behavior was not observed for any of them (Figure S24 and Supporting Information). In the case of the reference **ALP** inhibitor, we did not perform this analysis because the RMSD already indicated that its binding affinity was not in the same region as that of the other compounds.

**Figure 12:**
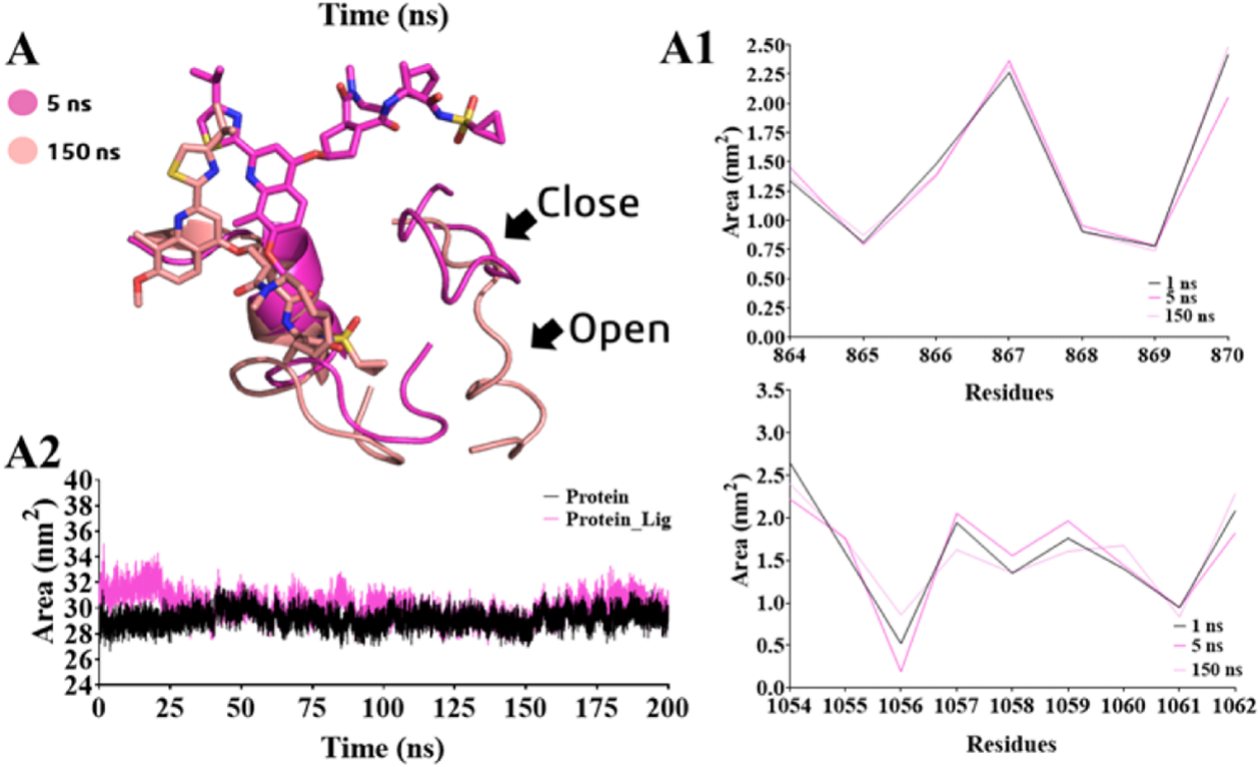
(A) Loop-1 (residues 851–870) and loop-2 (residues 1054–1062) in the presence of simeprevir (**SIM**) in two time periods. (A1) Variation in the area (nm^2^) of loop-1 and loop-2 related to the presence of **SIM**. (A2) Average solvent-accessible surface area (SASA, nm^2^) of residues 770–932, comprising the PI3Kα catalytic site.

Figure 13(A–E) shows the hydrogen bond (H-bond) lifetime (%) and representative H-bond interactions of PI3Kα in complex with ligands **CER** (A), **FUR** (B), **SIM** (C), **TRO** (D), and **VEM** (E). Among them, **FUR** exhibited the longest lifetime in H-bond interactions. Specifically, the formamide (carbonyl) group of **FUR** displayed a high persistence of 31 to 80% in its H-bond with the amino acid residue Ile771. Additionally, H-bond interactions of 14 to 19% were observed between the pyrimidine ring of the **FUR** ligand and residues Arg770 and His1060 (Figure 13B). Another compound, **SIM**, also demonstrated an intermediate lifetime H-bond interaction. It interacted via a hydrogen bond through the sulfonamide and amide (carbonyl) groups with Gln859, ranging from 12 to 37%, and the methoxyl group with Ser854, about 11% (Figure 13C).

**Figure 13:**
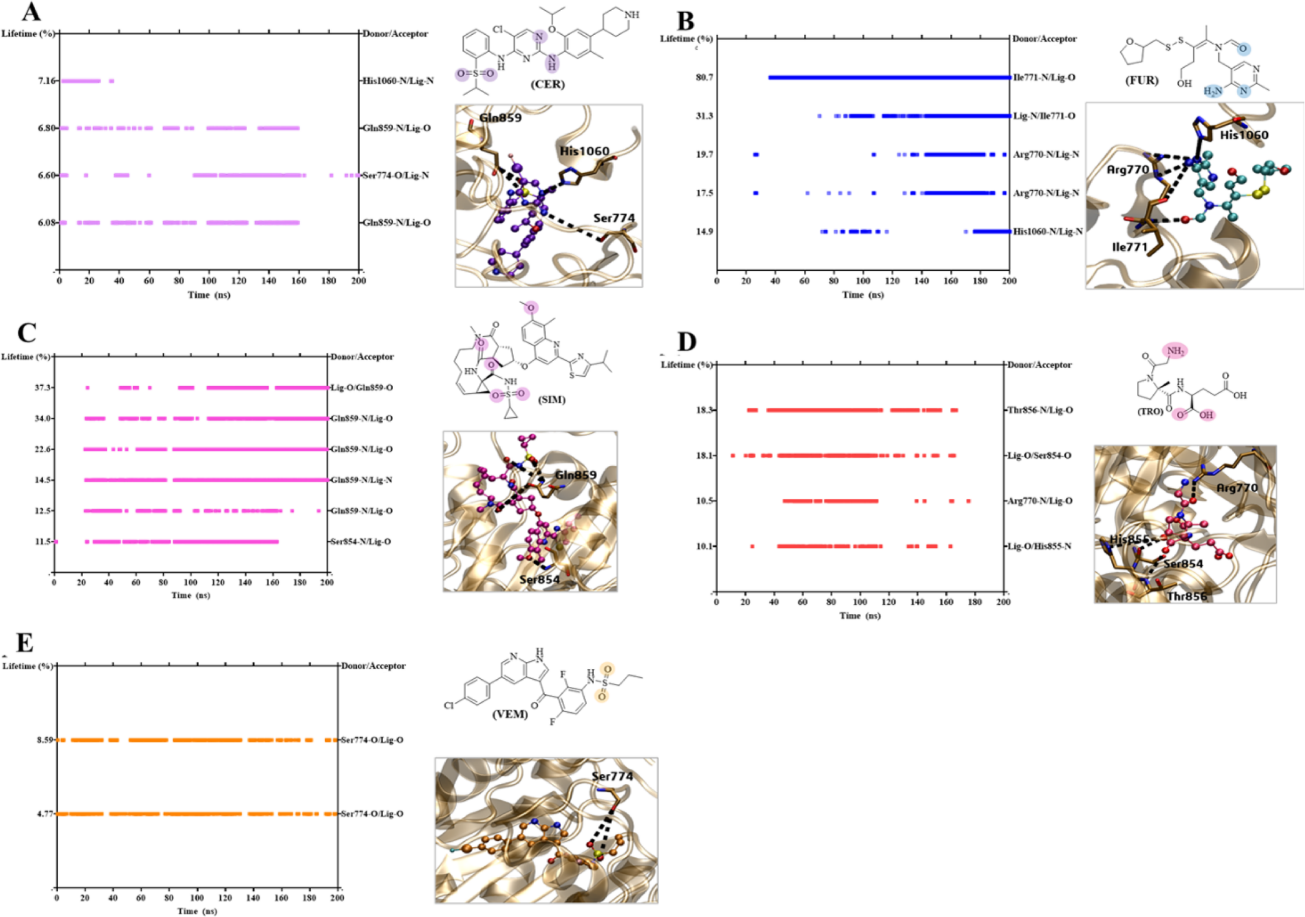
Hydrogen bond lifetime (%) and representative interactions of PI3Kα in complex with ligands (A) **CER**, (B) **FUR**, (C) **SIM**, (D) **TRO**, and (E) **VEM** during 200 ns of MDS. The colored circles indicate the atoms from ligands in interaction.

The **SIM** compound effectively maintained its H-bond interactions while moving around the ATP site. In contrast, the **TRO** compound did not exhibit the same H-bond patterns despite remaining stable in the protein’s active site, according to the RMSD analysis. It showed H-bond interactions between the amine and carboxyl groups with residues Thr856, Ser854, Arg770, and His855, with lifetimes varying from 10 to 18% (Figure 13D).

Finally, compounds **VEM** with its sulfonamide group and **CER** with its sulfonyl group and pyrimidine ring presented H-bonds with less persistence. **VEM** interacted via an H-bond only with residue Ser774 (4 to 8%) (Figure 13E), while **CER** interacted via H-bonds with Ser774, Gln859, and His1060, varying from 6 to 7% (Figure 13A).

We also conducted an H-bond analysis for the reference drug **ALP**. After 50 ns of simulation, this compound established interactions in a region near chain B (p85α), approximately 50 Å away from the kinase domain. The lifetimes of these interactions varied from 5 to 23% in H-bonds with Trp11, Arg244, and Asp240 (Figure S25, Supporting Information). Our results, both considering the number of bonds made by **ALP** (four H-bonds) and the calculated average RMSD value, were close to those reported in the literature.^99^ This indicates a propensity of this ligand to stabilize in this region.

The binding free energies (Δ*G*_bind_, kcal/mol) between the protein and each ligand, obtained from trajectories from the MDS of the protein–ligand complexes, were calculated by using the molecular mechanics/Poisson–Boltzmann surface area (MM/PBSA) method (Table 5). Therefore, the decreasing order of protein–ligand binding affinity was estimated from the calculated Δ*G*_bind_ values as follows (Table 5): **SIM** (Δ*G*_bind_ = −33.0 ± 3.2 kcal/mol) > **CER** (Δ*G*_bind_ = −25.2 ± 2.4 kcal/mol) > **FUR** (Δ*G*_bind_ = −14.5 ± 0.7 kcal/mol) > **ALP** (Δ*G*_bind_ = −12.0 ± 1.3 kcal/mol) > **TRO** (Δ*G*_bind_ = −8.63 ± 3.5 kcal/mol) > **VEM** (Δ*G*_bind_ = 128 ± 16 kcal/mol).

**Table 5:** Binding Free Energy (Δ*G*_bind_) and Contribution Terms (van der Waals, Δ*E*_vdW_; Electrostatic, Δ*E*_elect_; Solvation, Δ*E*_solv_; and Solvent Accessible Surface Area, Δ*E*_sasa_) of the PI3Kα–Ligand Complexes, Calculated for Alpelisib (**ALP**), Ceratinib (**CER**), Fursultiamine (**FUR)**, Simeprevir (**SIM**), Trofinetide (**TRO**), and Vemurafenib (**VEM**) with the MM/PBSA Method (Mean ± Standard Deviation Energies; Kcal/Mol)

Ligand	Δ*G*_bind_	Δ*E*_vdW_	Δ*E*_elect_	Δ*E*_solv_	Δ*E*_sasa_
**ALP**	–12.0 ± 1.3	–28.1 ± 1.7	–14.1 ± 4.0	33.8 ± 3.7	–3.56 ± 0.1
**CER**	–25.2 ± 2.4	–39.6 ± 2.0	–2.1 ± 0.8	21.4 ± 2.4	–4.91 ± 0.2
**FUR**	–14.5 ± 0.7	–19.5 ± 1.5	–1.29 ± 1.7	8.52 ± 2.7	–2.24 ± 0.2
**SIM**	–33.0 ± 3.2	–61.5 ± 2.4	–4.84 ± 1.9	40.4 ± 2.5	–7.10 ± 0.2
**TRO**	–8.63 ± 3.5	–25.8 ± 1.1	–21.8 ± 1.3	42.8 ± 2.8	–3.82 ± 0.1
**VEM**	128 ± 16	–8.97 ± 16	–15.7 ± 11	158 ± 8.5	–4.70 ± 0.1

Based on the binding free energy analysis, it is indicated that the hydrophobicity of some ligands, e.g., **SIM** and **CER**, is favorable for the binding free energy contributions. This is probably due to the hydrophobic characteristics of several residues in the kinase domain, i.e., glycine, serine, tryptophan, isoleucine, methionine, phenylalanine, proline, and valine.

As a result, we observed a high contribution of the van der Waals energy term (Δ*E*_vdW_ = −61.5 ± 2.4 kcal/mol) for **SIM**, which was not compensated by the solvation energy term (Δ*E*_solv_ = 40.4 ± 2.5 kcal/mol). This led to the best result of binding affinity for **SIM** (Δ*G*_bind_ = −33.0 ± 3.2 kcal/mol).

Like the PI3Kα-**SIM** complex but showing twice smaller contributions of the van der Waals, electrostatic, and solvation energy terms, the formation of the PI3Kα–**CER** complex was favorable, resulting in the second-best binding affinity (Δ*G*_bind_ = −25.2 ± 2.4 kcal/mol) (Table 5).

**FUR** has a more hydrophilic structural profile than the last two compounds mentioned (**SIM** and **CER**), presenting a less required solvation energy (Δ*E*_solv_ = 8.52 ± 2.7 kcal/mol), resulting in a higher binding affinity value observed (Δ*G*_bind_ = −14.5 ± 0.7 kcal/mol). **TRO** has two carboxyl and one amine group that are possibly ionizable in aqueous systems, resulting in a more significant contribution of the electrostatic energy term (Δ*E*_elect_ = −21.8 ± 1.3 kcal/mol) than other compounds.

This also increases the energetic cost of solvation, resulting in its higher binding affinity value calculated (Δ*G*_bind_ = −8.63 ± 3.5 kcal/mol). The binding free energy profile of **TRO** is noteworthy. It has been found that the four energy terms (i.e., Δ*E*_vdW_, Δ*E*_elect_, Δ*E*_solv_, and Δ*E*_sasa_) are like those of the reference drug **ALP**. Additionally, the binding free energy of **TRO** (Δ*G*_bind_ = −8.63 ± 3.5 kcal/mol) is comparable to that of **ALP** (ΔG_bind_ = −12.0 ± 1.3 kcal/mol) (Table 5).

The high solvation energy cost (Δ*E*_solv_ = 158 ± 8.5 kcal/mol) of **VEM** in interaction with PI3Kα results in an unfavorable binding affinity (Δ*G*_bind_ = 128 ± 16 kcal/mol), indicating the weaker potential of this compound in forming a stable complex and acting as a possible inhibitor (Table 5).

In summary, except for the **VEM** compound and within the limitations of the structural constraints of the other four compounds assessed, they consistently demonstrate a great affinity for the molecular target PI3Kα. This suggests their potential to act as competitive inhibitors of this protein. Our analysis through molecular dynamics has indicated robust stability of interaction in the kinase domain, where the catalytic residues are located, opening the door for in vitro studies.

## Conclusions

In this study, we employed a hybrid computational strategy, integrating machine learning and molecular modeling techniques, to identify potential PI3Kα inhibitors from the DrugBank/FDA-approved drugs data set for drug repurposing. Our random forest classification model, combined with molecular docking, 200 ns molecular dynamics simulations, and MM/PBSA binding free energy calculations, identified five candidates. The results identified simeprevir (Δ*G*_bind_ = – 33.0 ± 3.2 kcal/mol) and ceritinib (ΔG_bind_ = −25.2 ± 2.4 kcal/mol) as the most promising candidates, demonstrating favorable estimated binding affinities and stable interactions within the kinase domain of the PI3Kα enzyme. Beyond these findings, this work also contributes to an interactive and publicly accessible tool, the “PI3Kα Activity Predictor”. This application provides the validated machine learning (ML) model and incorporates reliability (applicability domain) checks, which are important components for the practical application of predictive models. We acknowledge that the primary limitation of this study is that the results are entirely computational. In the future, we intend to perform experimental validation to confirm these findings. This study, therefore, provides the rationale and theoretical basis for *in vitro* and *in vivo* assays, which are necessary to confirm the therapeutic potential of simeprevir and ceritinib in treating conditions associated with PI3Kα dysregulation such as post-CoViD-19 pulmonary fibrosis.

## Supplementary Material



## Data Availability

The data used in the random forest (RF) model construction and validation were obtained from ChEMBL. An application (app) to make predictions for PI3Kα bioactivity named “PI3Kα Activity Predictor” is publicly available on GitHub (https://github.com/carineribeirost/pi3k-streamlit-app). The “PI3Kα Activity Predictor” app developed by C.R.S. is a Streamlit application that predicts PI3Kα activity based on molecular SMILES and visualizes the applicability domain (AD) of the predictions.

## Notes

It contains the top 10 models by balanced accuracy using Lazy Predict Python library (Figure S1); the validation results for the 3D model refined by molecular dynamics simulation (MDS) and the comparison with the experimental 3D structures (PDB ID: 4JPS 4YKN and 5DXH) used as templates (Figures S2–S6, Verify 3D plots; Figures S7–S16, ERRAT plots; Figures S17–S23, Ramachandran plots); and the summarized SAVES (v.6.0) results (ERRAT, 3D-1D score, Verify 3D) for the 3D structures of the PDB templates (4JPS, 4YKN, and 5DXH) and the model refined by MDS (**Table S1**); while Figure S24 shows the loop-1 and loop-2 behavior from MDS of complex with **FUR**, **CER**, and **VEM**; and Figure S25 shows the H-bond lifetime from MDS of complex with **ALP** (PDF)
