# Supplementary material for: Machine Learning and Molecular Modeling for Drug Repurposing Targeting Potential PI3Kα Inhibitors in Post-CoViD-19 Pulmonary Fibrosis
Source: ACS Omega. 2026 Jan 1;11(2):2868–87. doi: 10.1021/acsomega.5c08980 (PMC12824809; doi:10.1021/acsomega.5c08980)
Supplement: Supplementary file 1 [file ao5c08980_si_001.pdf]

Supporting Information

Machine learning and molecular modeling for drug repurposing targeting potential PI3Kα inhibitors in post-CoViD-19 pulmonary fibrosis

Carine Ribeiro dos SANTOS [1], **Priscila Goes CAMARGO** [1,2], **Carlos Rangel RODRIGUES** [2], **Camilo Henrique da Silva LIMA** *[1], **Magaly Girão ALBUQUERQUE** *[1]

1) Universidade Federal do Rio de Janeiro (UFRJ), Centro de Ciências Matemáticas e da Natureza (CCMN), Instituto de Química (IQ), Departamento de Química Orgânica (DQO), Programa de Pós-Graduação em Química (PGQu), Laboratório de Modelagem Molecular Prof. Ricardo Bicca de Alencastro (LabMMol), Avenida Athos da Silveira Ramos, nº 149, Centro de Tecnologia, Bloco A, Cidade Universitária, Rio de Janeiro, RJ, CEP 21941-909, Brazil.

2) Universidade Federal do Rio de Janeiro (UFRJ), Centro de Ciências da Saúde (CCS), Faculdade de Farmácia (FF), Departamento de Fármacos e Medicamentos (DEFARMED), Laboratório de Modelagem Molecular & QSAR (ModMolQSAR), Avenida Carlos Chagas Filho, 373, Cidade Universitária, Rio de Janeiro, RJ, CEP 21941-902, Brazil.

This PDF file "**Supporting Information**" is available free of charge. It contains the top 10 models by balanced accuracy using Lazy Predict Python library (**Figure S1**); the validation results for the 3D model refined by molecular dynamics simulation (MDS) and the comparison with the experimental 3D structures (PDB ID: 4JPS, 4YKN, and 5DXH) used as templates (**Figure S2–S6**, Verify 3D plots; **Figure S7–S16**, ERRAT plots; **Figure S17–S23**, Ramachandran plots); and the summarized SAVES (v.6.0) results (ERRAT, 3D-1D score, Verify 3D) for the 3D structures of the PDB templates (4JPS, 4YKN, and 5DXH) and the model refined by MDS (**Table S1**); while **Figure S24** shows the loop-1 and loop-2 behavior from MDS of complex with **FUR**, **CER**, and **VEM**; and **Figure S25** shows the H-bond lifetime from MDS of complex with **ALP**.

Figure S1. Top 10 models by balanced accuracy using Lazy Predict Python library………..………… **S3**

Figure S2. Verify 3D plots for 4JPS template (chain A and chain B)…………………………………. **S4**

Figure S3. Verify 3D plot for 4YKN template………………………………………………………. **S5**

Figure S4. Verify 3D plots for 5DXH template (chain A and chain B)……………………………... **S6**

Figure S5. Verify 3D plots for 5DXH template (chain D and chain E)……………………………... **S7**

Figure S6. Verify 3D plots for the model (chain A and chain B) refined by MDS……………….…... **S8**

Figure S7. ERRAT plot for 4JPS template (chain A)……………………………………………….. **S9**

Figure S8. ERRAT plot for 4JPS template (chain B)……………………………………………….. **S10**

Figure S9. ERRAT plot for 4YKN template (chain A – 1)………………………………………….. **S11**

Figure S10. ERRAT plot for 4YKN template (chain A – 2)………………………………………….. **S12**

Figure S11. ERRAT plot for 5DXH template (chain A)…………………………………………….. **S13**

Figure S12. ERRAT plot for 5DXH template (chain B)…………………………………………….. **S14**

Figure S13. ERRAT plot for 5DXH template (chain D)…………………………….……………… **S15**

Figure S14. ERRAT plot for 5DXH template (chain E)…………………………………………….. **S16**

Figure S15. ERRAT plot for the model (chain A) refined by MDS……………………….………….. **S17**

Figure S16. ERRAT plot for the model (chain B) refined by MDS…………………………………... **S18**

Figure S17. Ramachandran plot for 4JPS template……………………………………………….. **S19**

Figure S18. Ramachandran plot for 4YKN template……………………………………………... **S20**

Figure S19. Ramachandran plot for 5DXH template……………………………………………... **S21**

Figure S20. Ramachandran plot for the model refined by MDS…………………………………… **S22**

Figure S21. Ramachandran Plot for 4JPS template………………………………………………. **S23**

Figure S22. Ramachandran plot for 4KYN template……………………………………………... **S24**

Figure S23. Ramachandran plot for 5DXH template……………………………………………... **S25**

Figure S24. Loop-1 (dark blue, purple, or orange color, 851–870) and loop-2 (light blue, purple, or orange color, 1054–1062) in the presence of (**A1**) **FUR**, (**B1**) **CER** and (**C1**) **VEM** in two time periods. Variation in the volume (nm^2) of loop-1 and loop-2 related to the presence of (**A2**) **FUR**, (**B2**) **CER**, and (**C2**) **VEM**. Average solvent accessible surface area (SASA) of residues 770–932, comprising the catalytic site of the PI3Kα kinase domain of (**A3**) **FUR**, (**B3**) **CER**, and (**C3**) **VEM**…………………………………… **S26**

Figure S25. H-bond lifetime (%) and representative interactions of **ALP**-PI3Kα complex during 200 ns of MDS. The colored circles indicated the atoms from ligands in interaction……………….……………... **S27**

Table S1. SAVES (v.6.0) results (ERRAT, 3D-1D score, and Verify 3D) for the 3D structures of the PDB templates (4JPS, 4YKN, and 5DXH) and the model refined by MDS……………………….…………….. **S28**

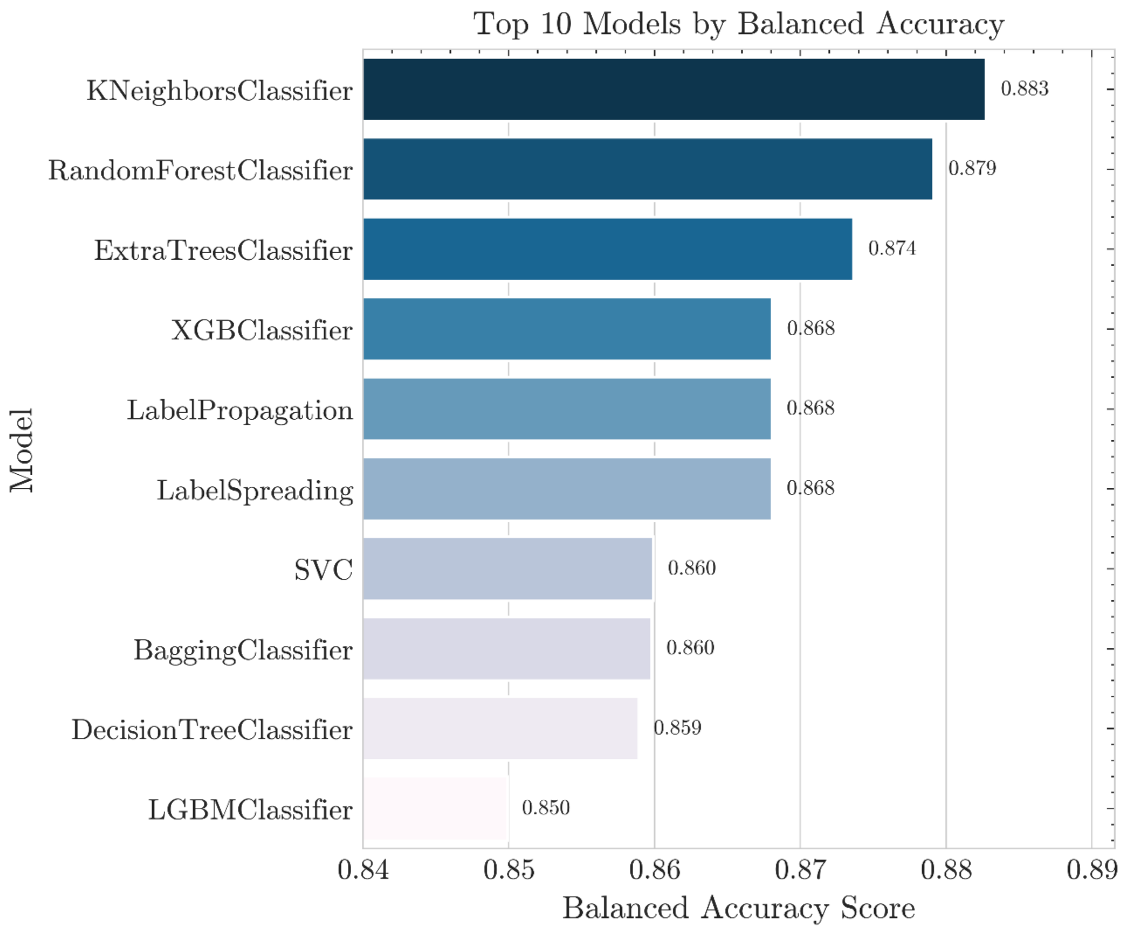

Figure S1. Top 10 models by balanced accuracy using Lazy Predict Python library.

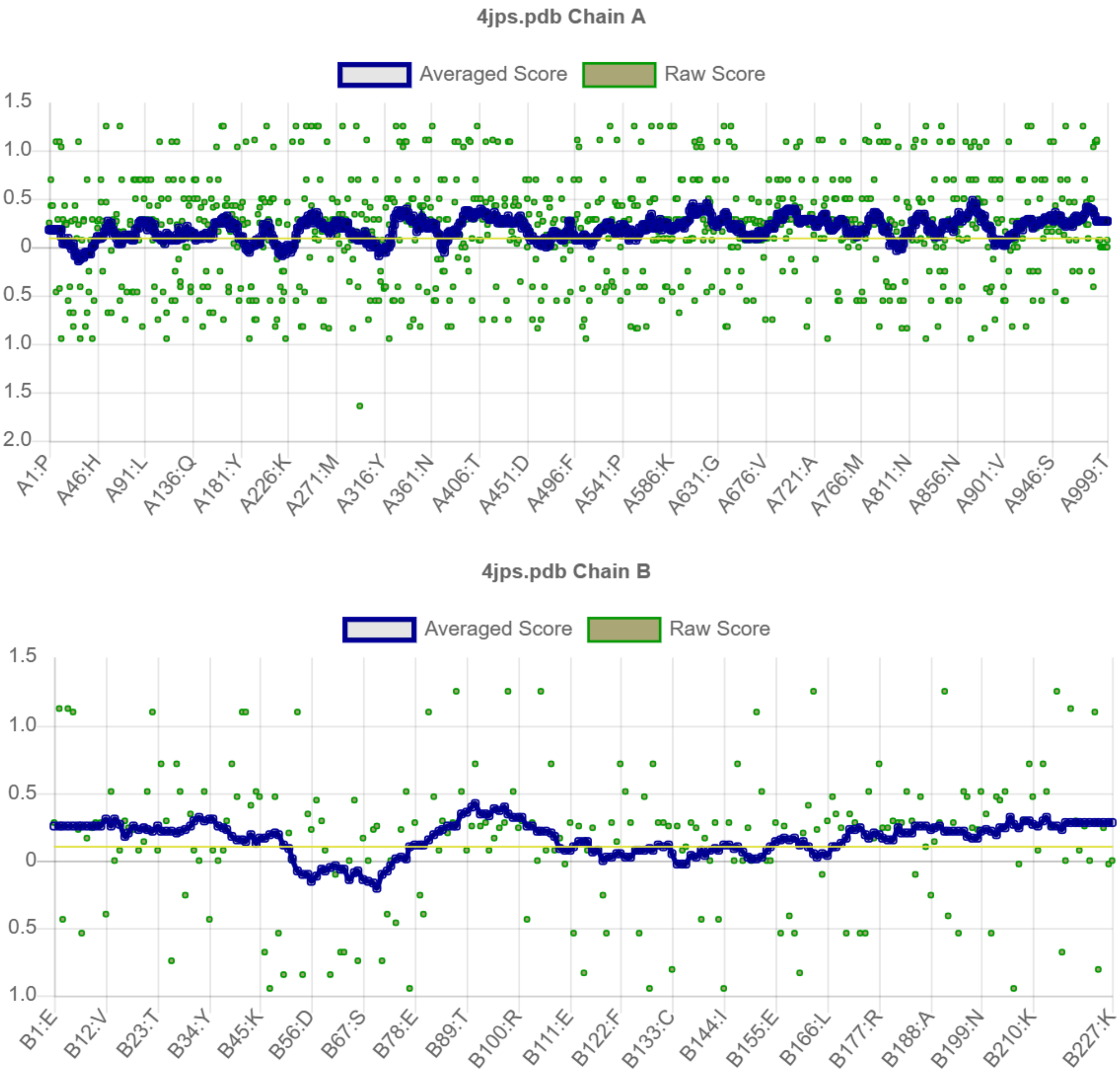

Figure S2. Verify 3D plots for 4JPS template (chain A and chain B).

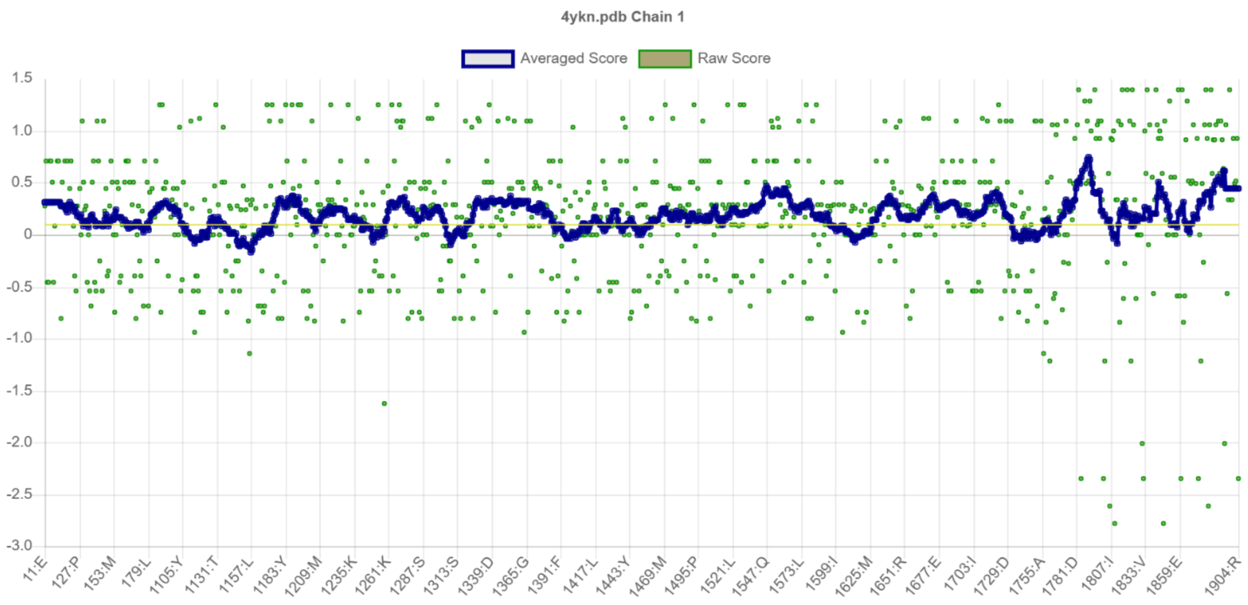

Figure S3. Verify 3D plot for 4YKN template (chain 1).

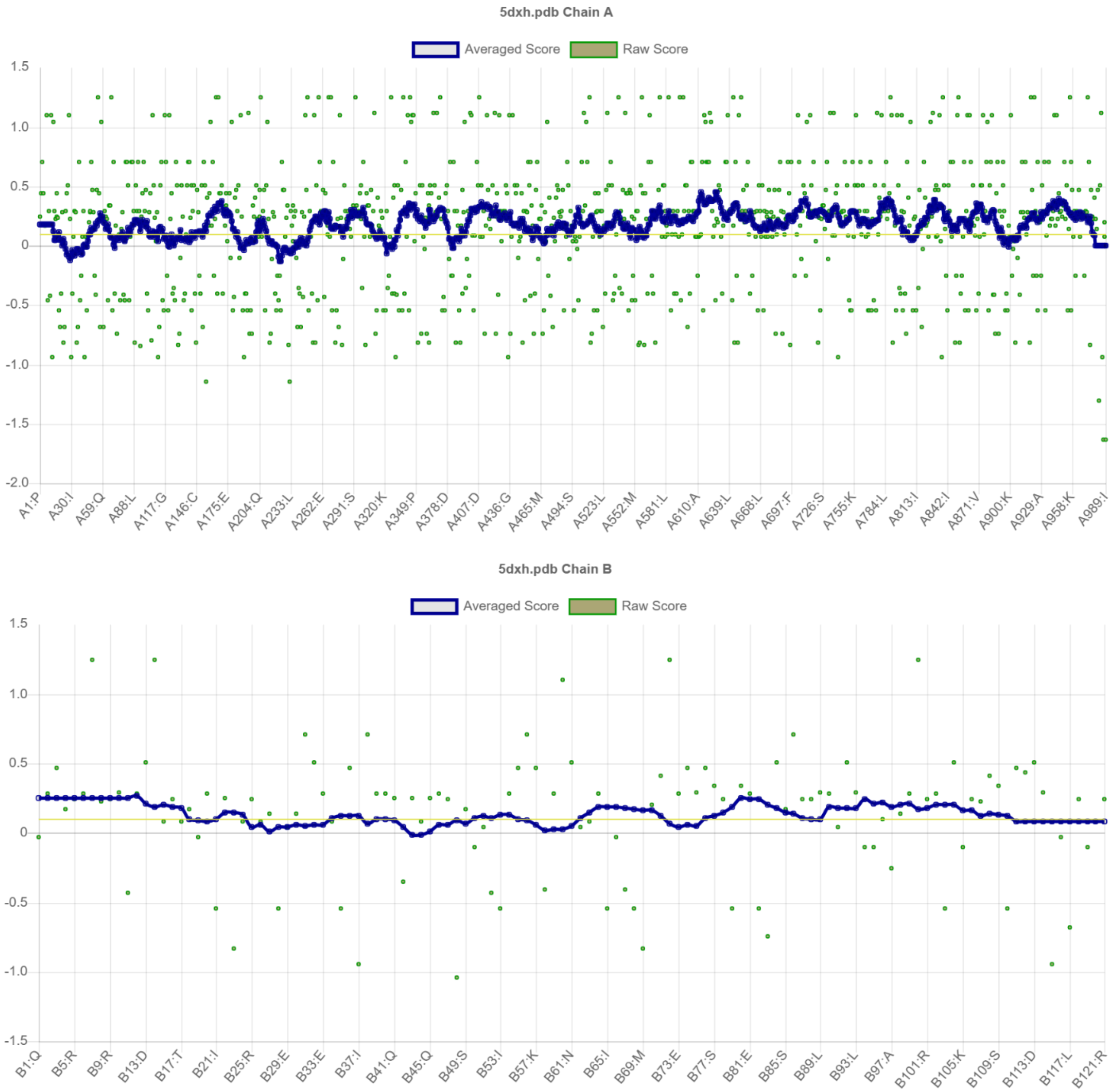

Figure S4. Verify 3D plots for 5DXH template (chain A and chain B).

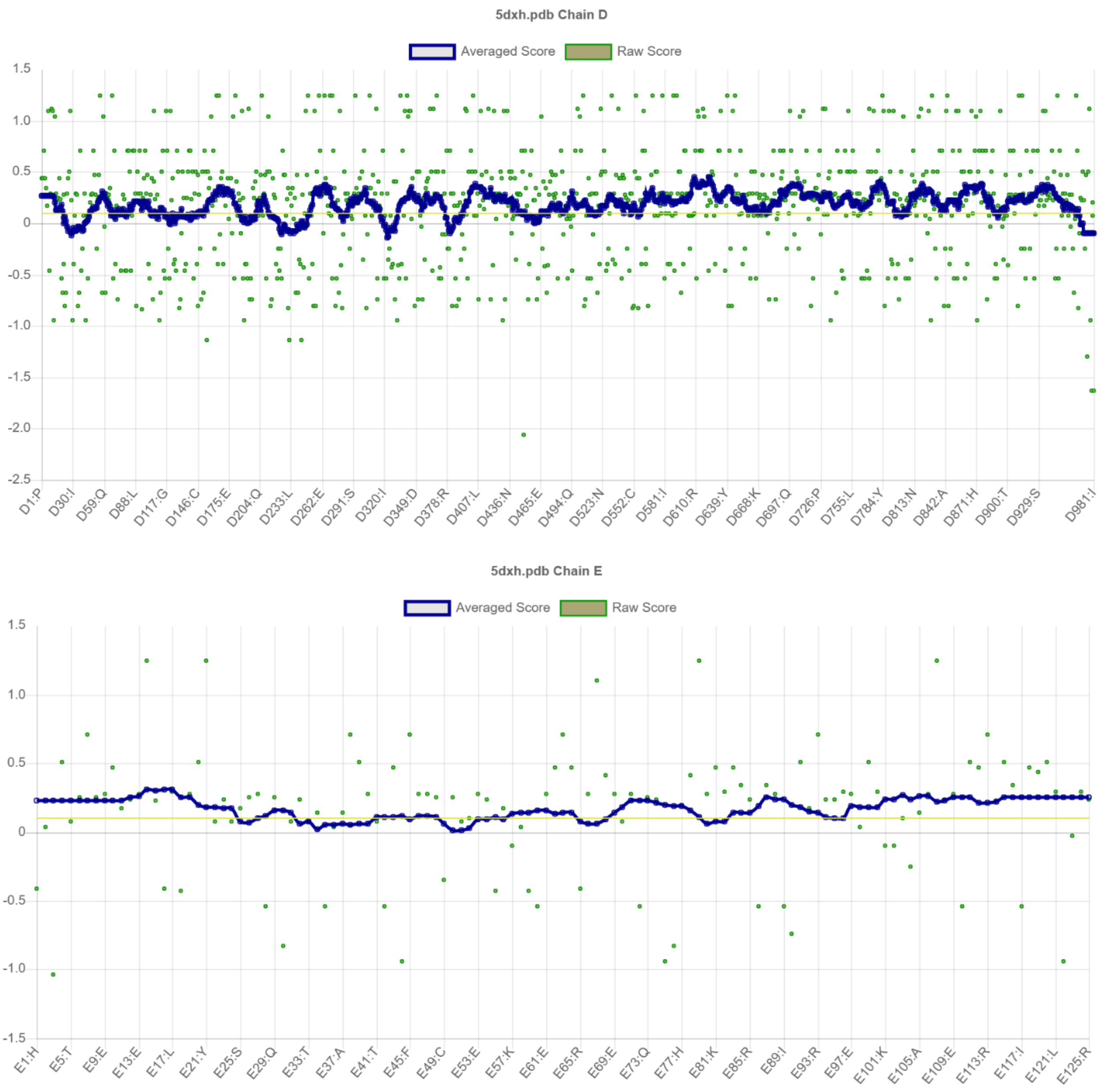

Figure S5. Verify 3D plots for 5DXH template (chain D and chain E).

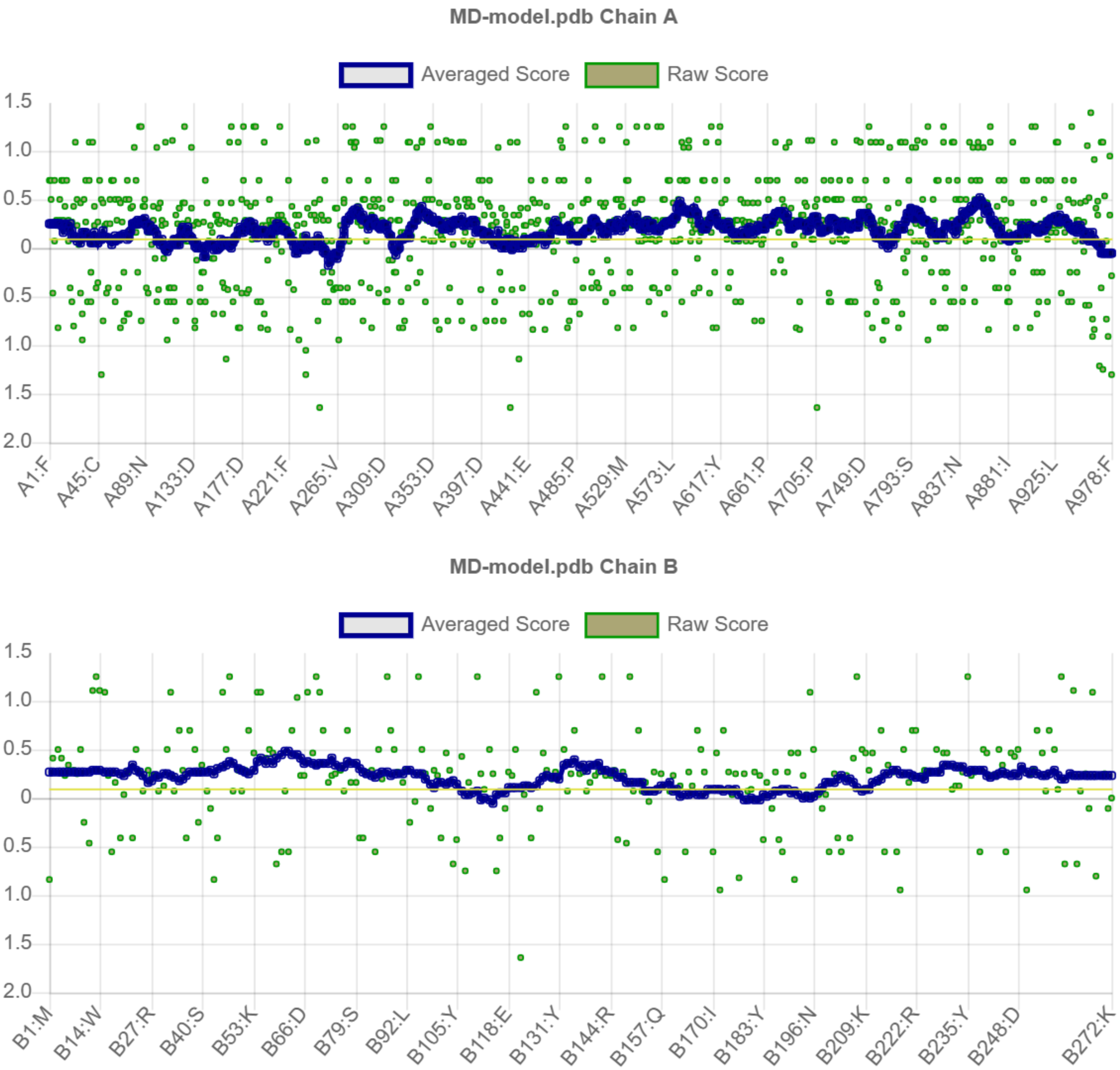

Figure S6. Verify 3D plots for the model (chain A and chain B) refined by MDS.

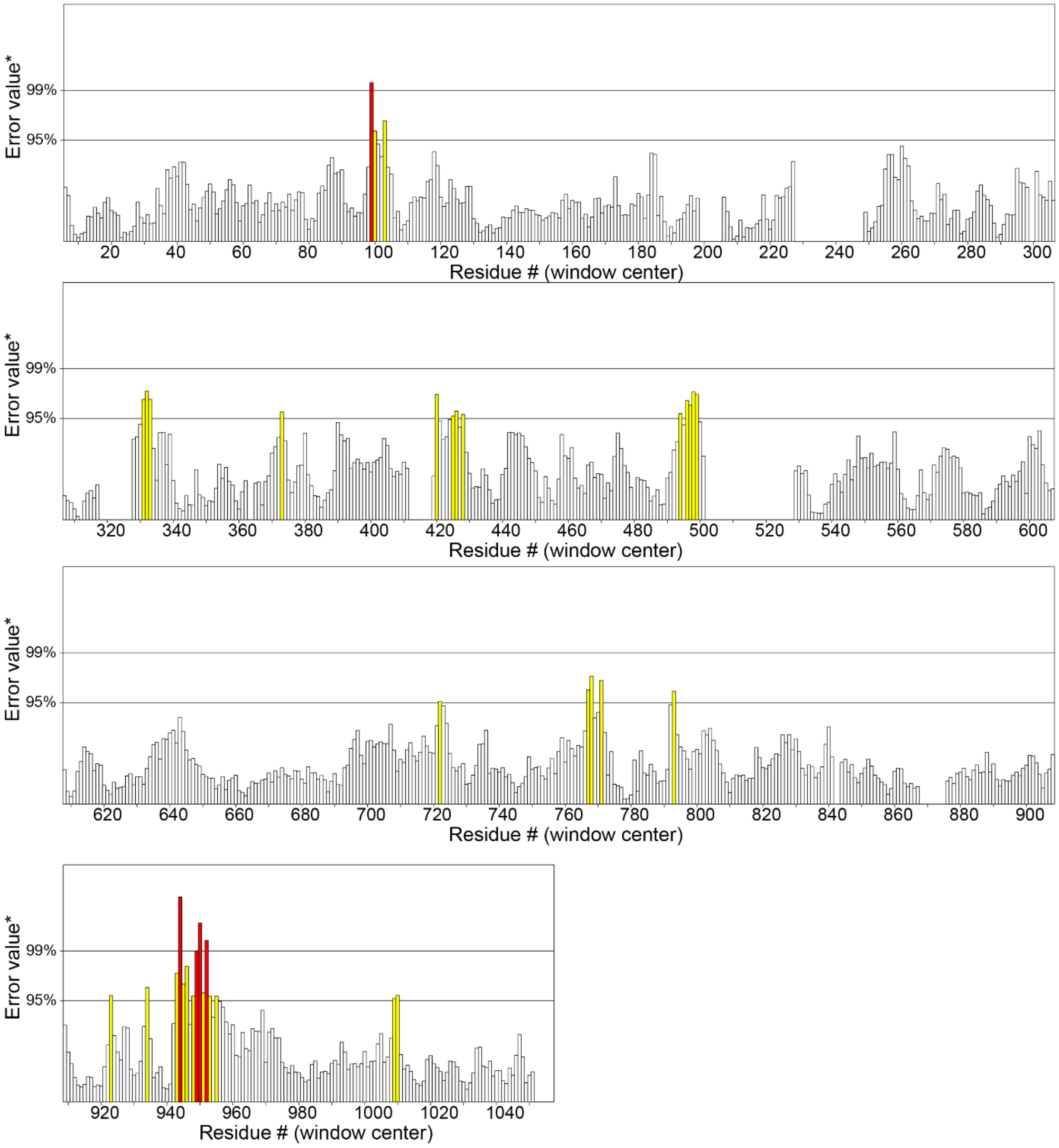

Figure S7. ERRAT plot for 4JPS template (chain A).

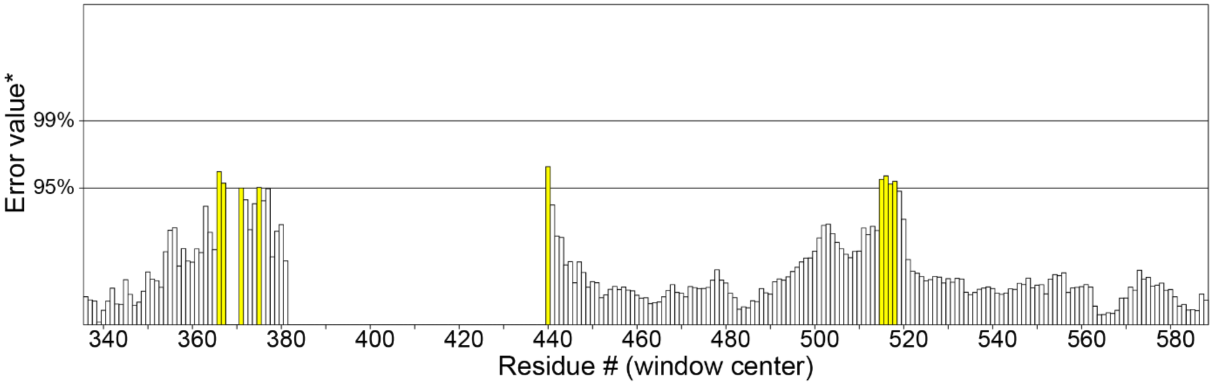

Figure S8. ERRAT plot for 4JPS template (chain B).

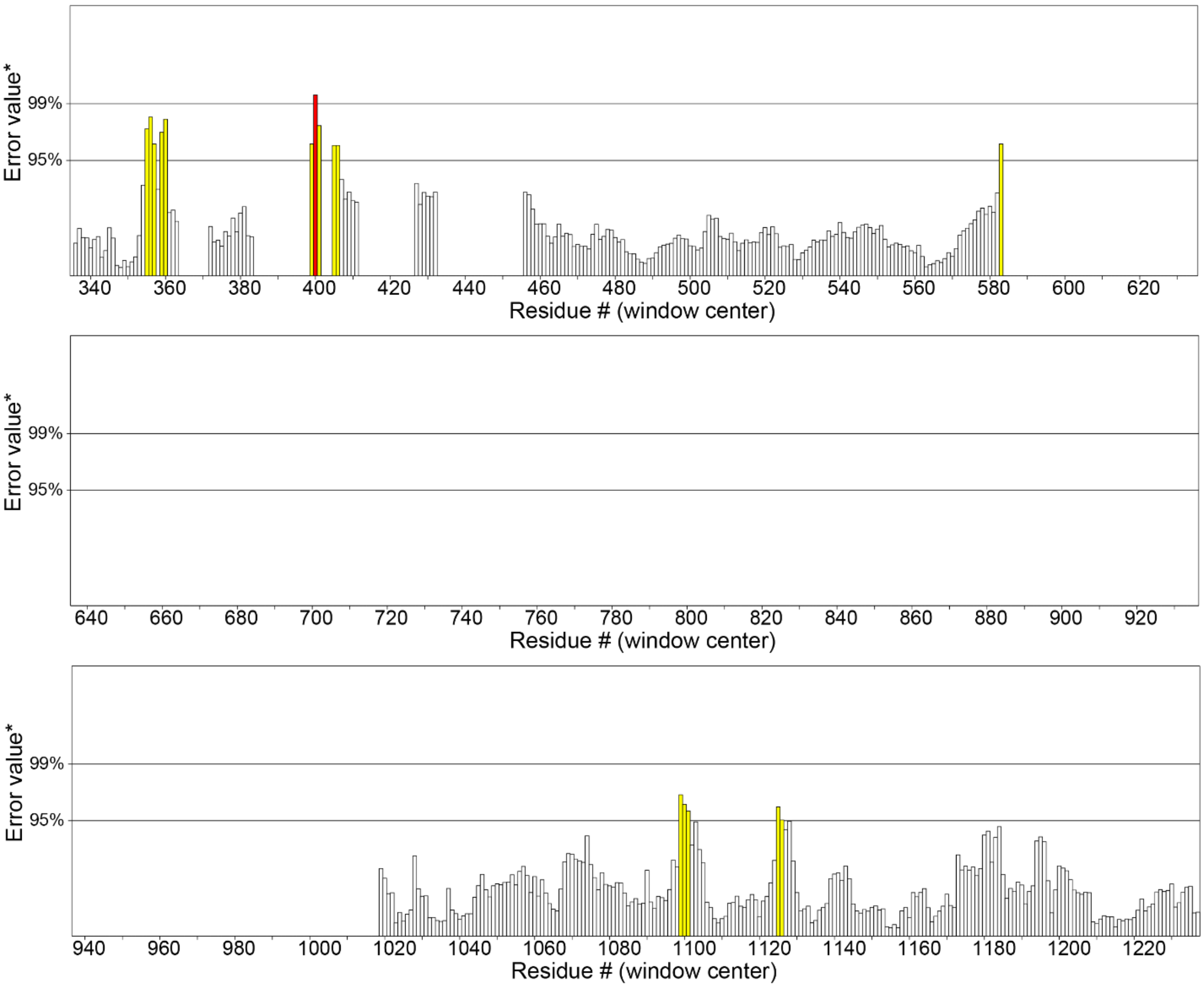

Figure S9. ERRAT plot for 4YKN template (chain A – 1).

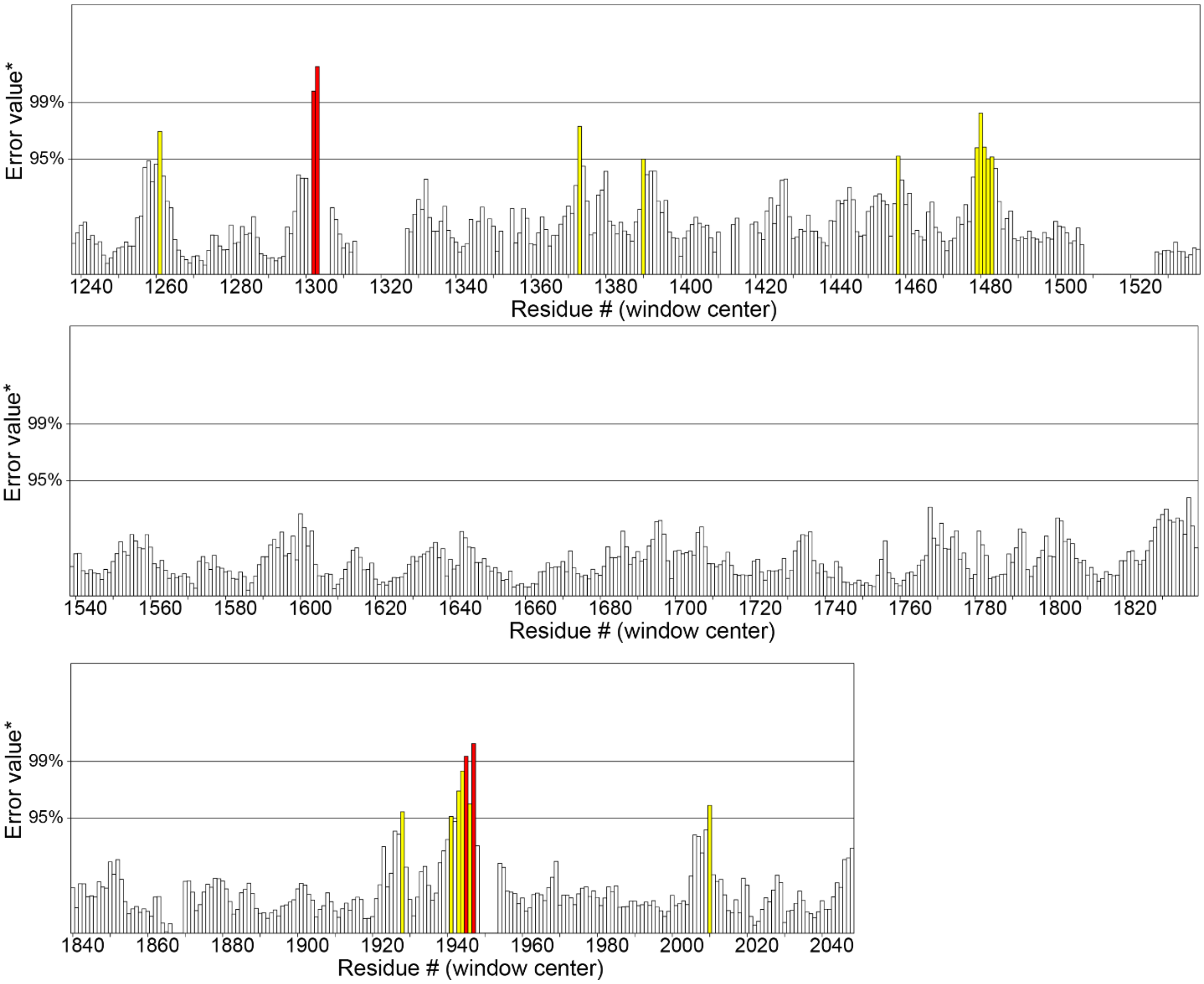

Figure S10. ERRAT plot for 4YKN template (chain A – 2).

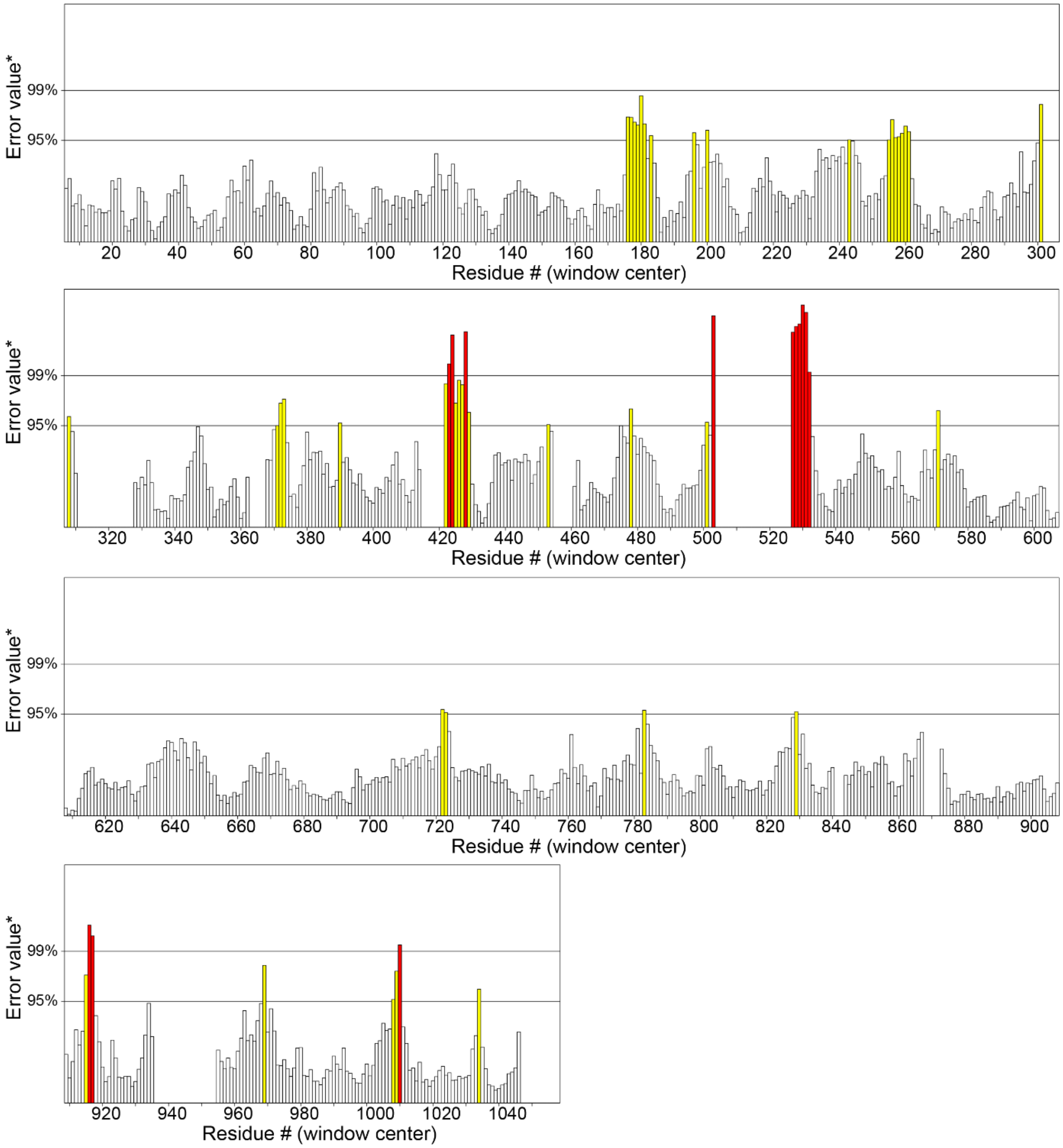

Figure S11. ERRAT plot for 5DXH template (chain A).

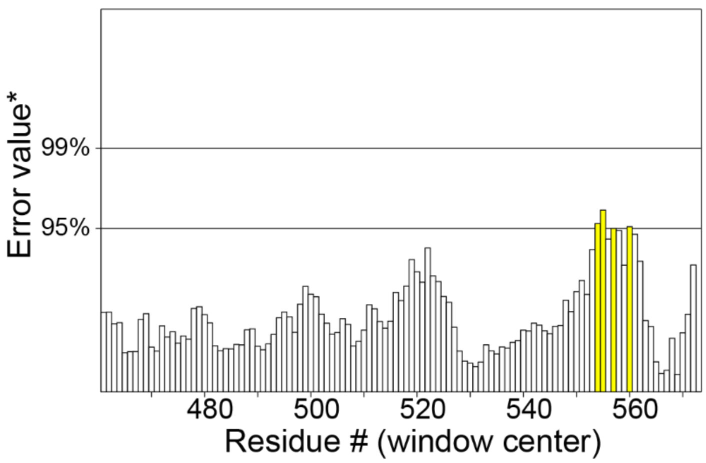

Figure S12. ERRAT plot for 5DXH template (chain B).

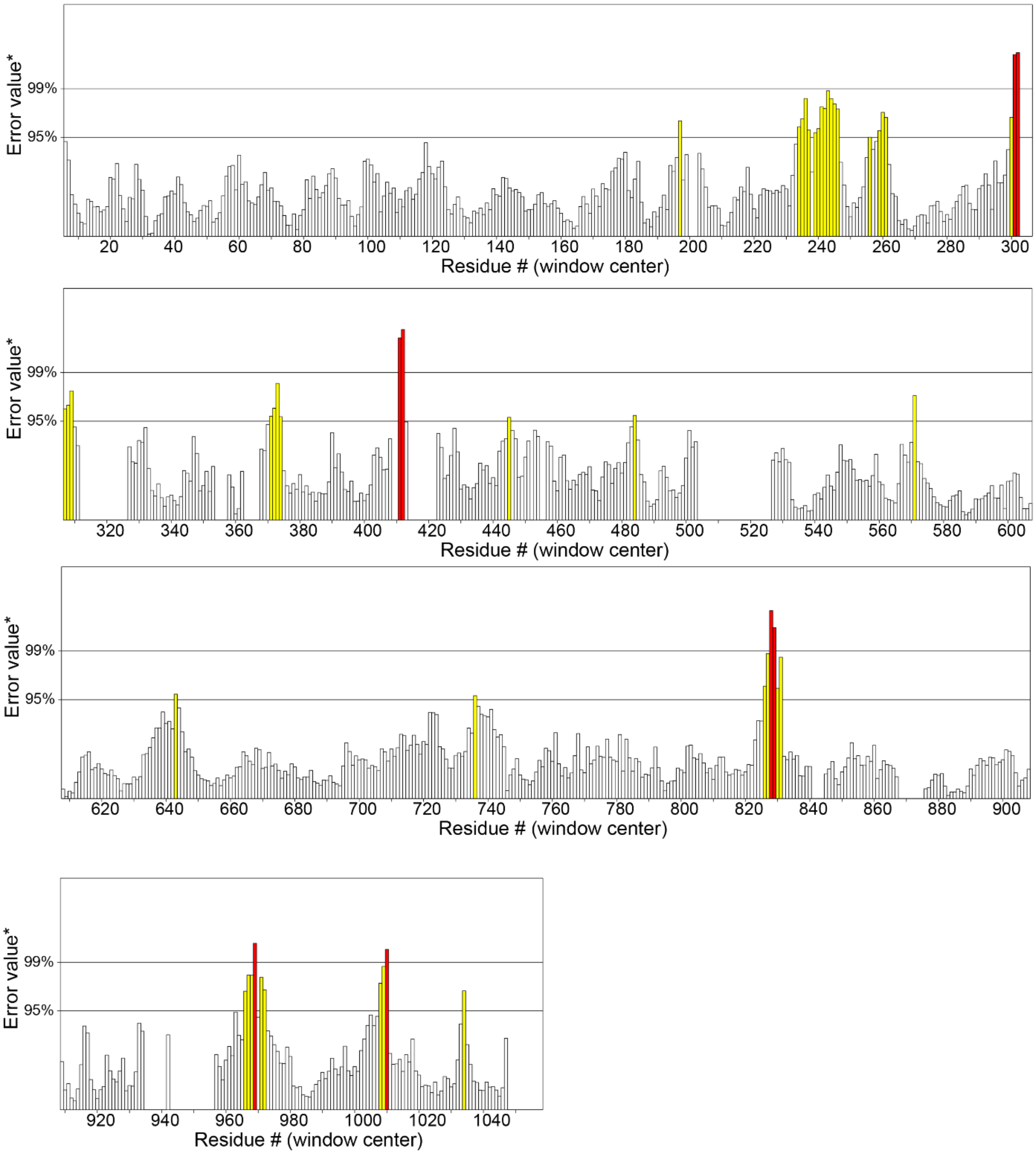

Figure S13. ERRAT plot for 5DXH template (chain D).

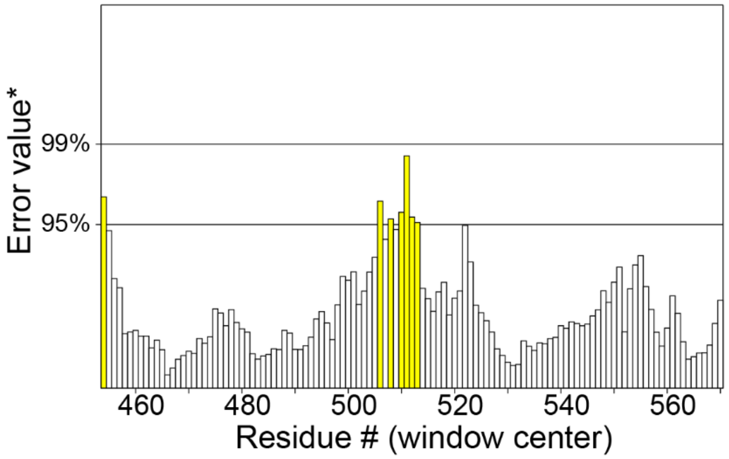

Figure S14. ERRAT plot for 5DXH template (chain E).

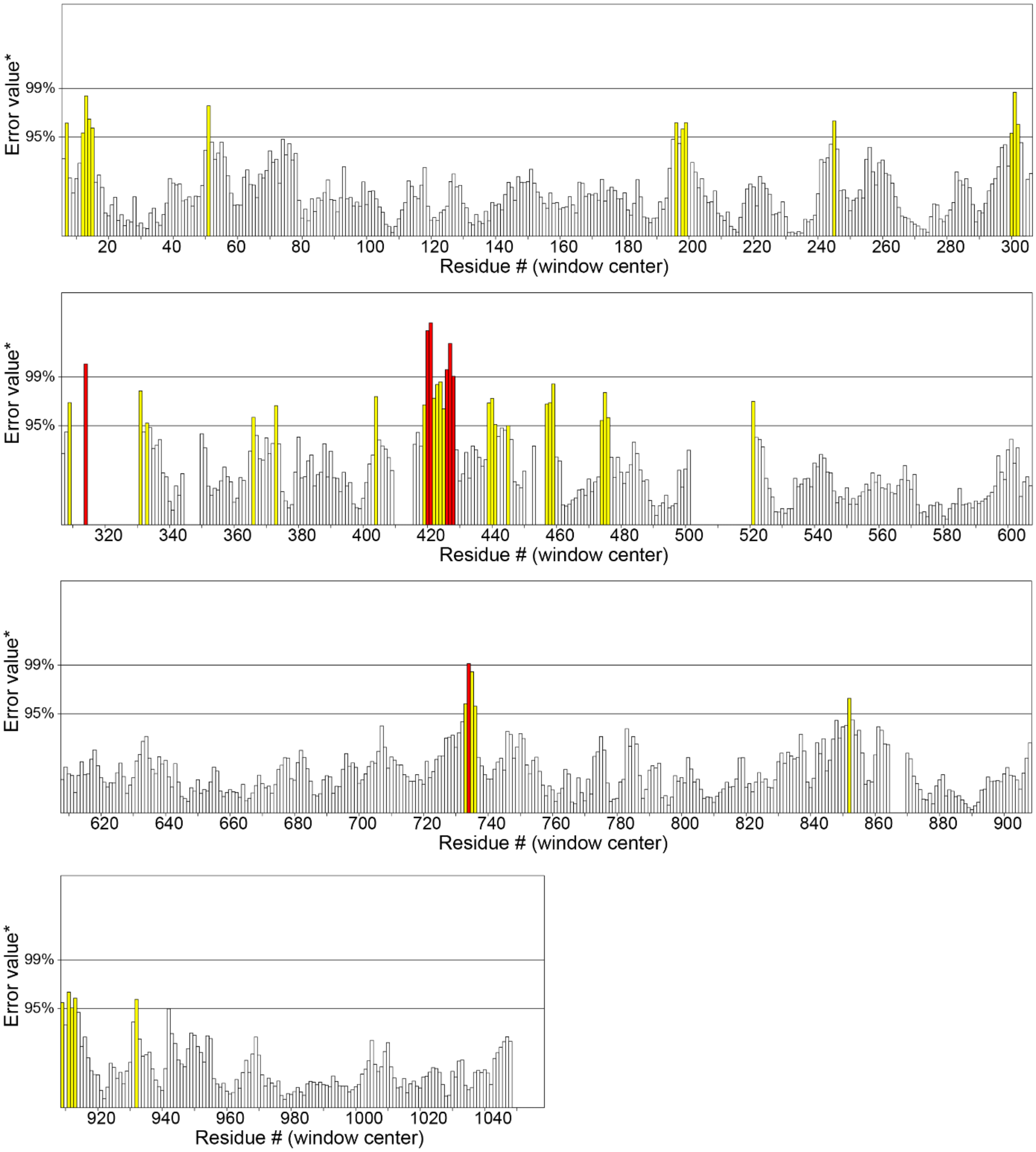

Figure S15. ERRAT plot for the model (chain A) refined by MDS.

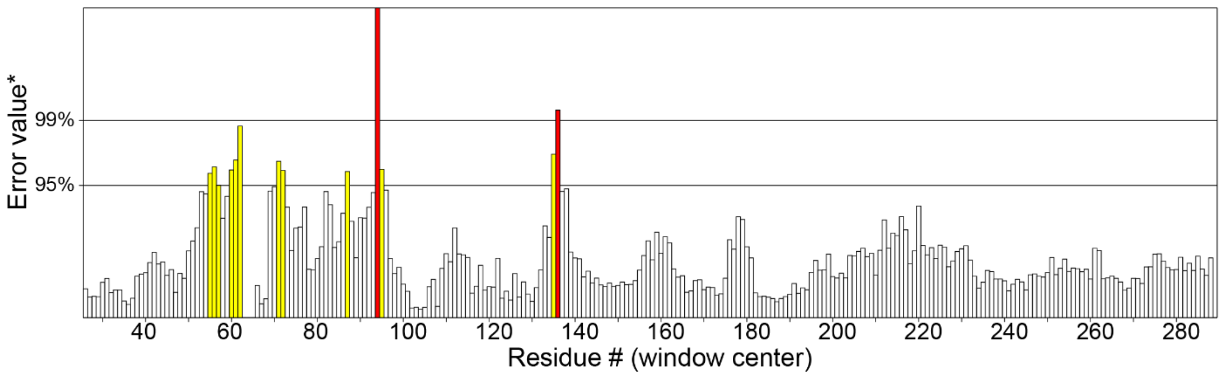

Figure S16. ERRAT plot for the model (chain B) refined by MDS.

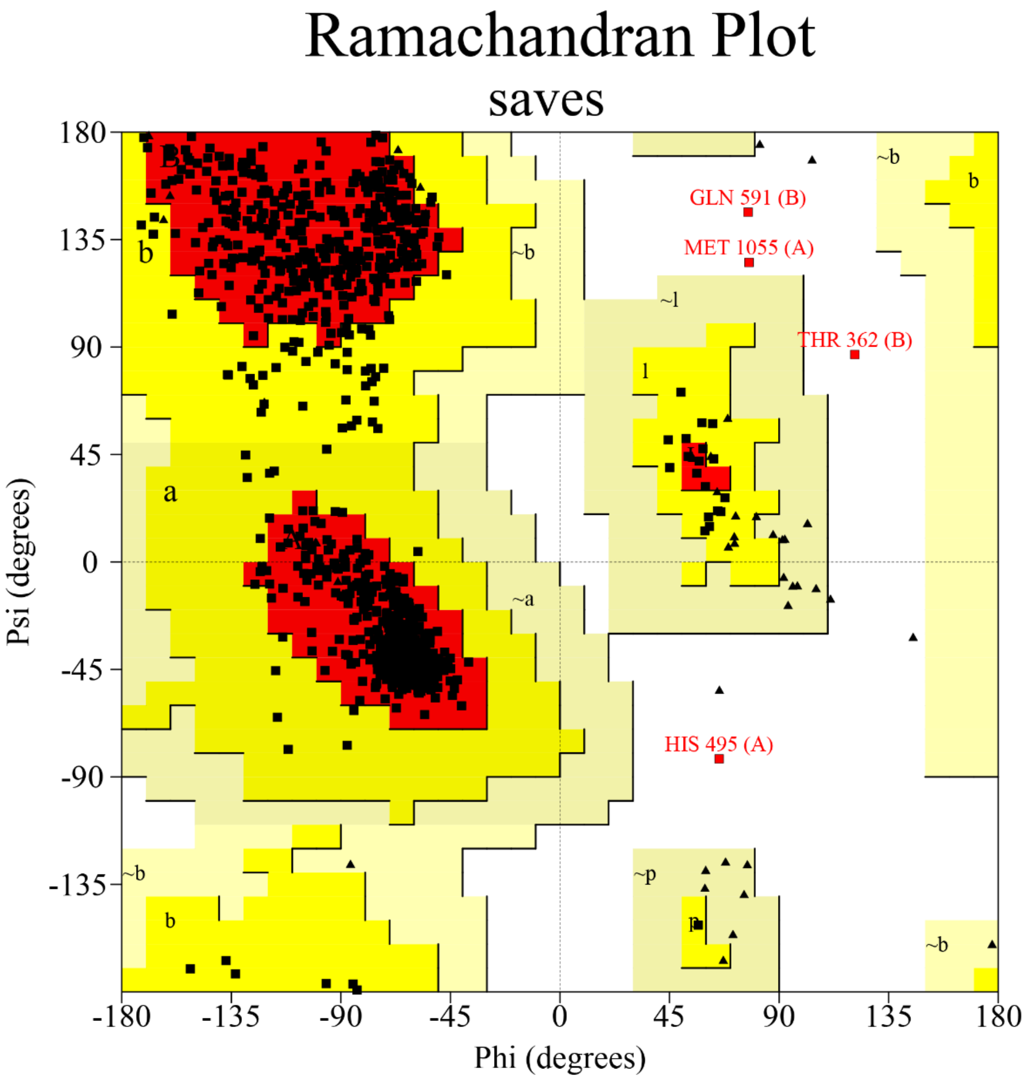

Plot statistics

Residues in most favoured regions [A,B,L]	1022	92.6%
Residues in additional allowed regions [a,b,l,p]	78	7.1%
Residues in generously allowed regions [~a,~b,~l,~p]	0	0.0%
Residues in disallowed regions	4	0.4%
	----	------
Number of non-glycine and non-proline residues	1104	100.0%
Number of end-residues (excl. Gly and Pro)	18	
Number of glycine residues (shown as triangles)	51	
Number of proline residues	53	

Total number of residues	1226	

Based on an analysis of 118 structures of resolution of at least 2.0 Angstroms and R-factor no greater than 20%, a good quality model would be expected to have over 90% in the most favoured regions.

Figure S17. Ramachandran plot for 4JPS template.

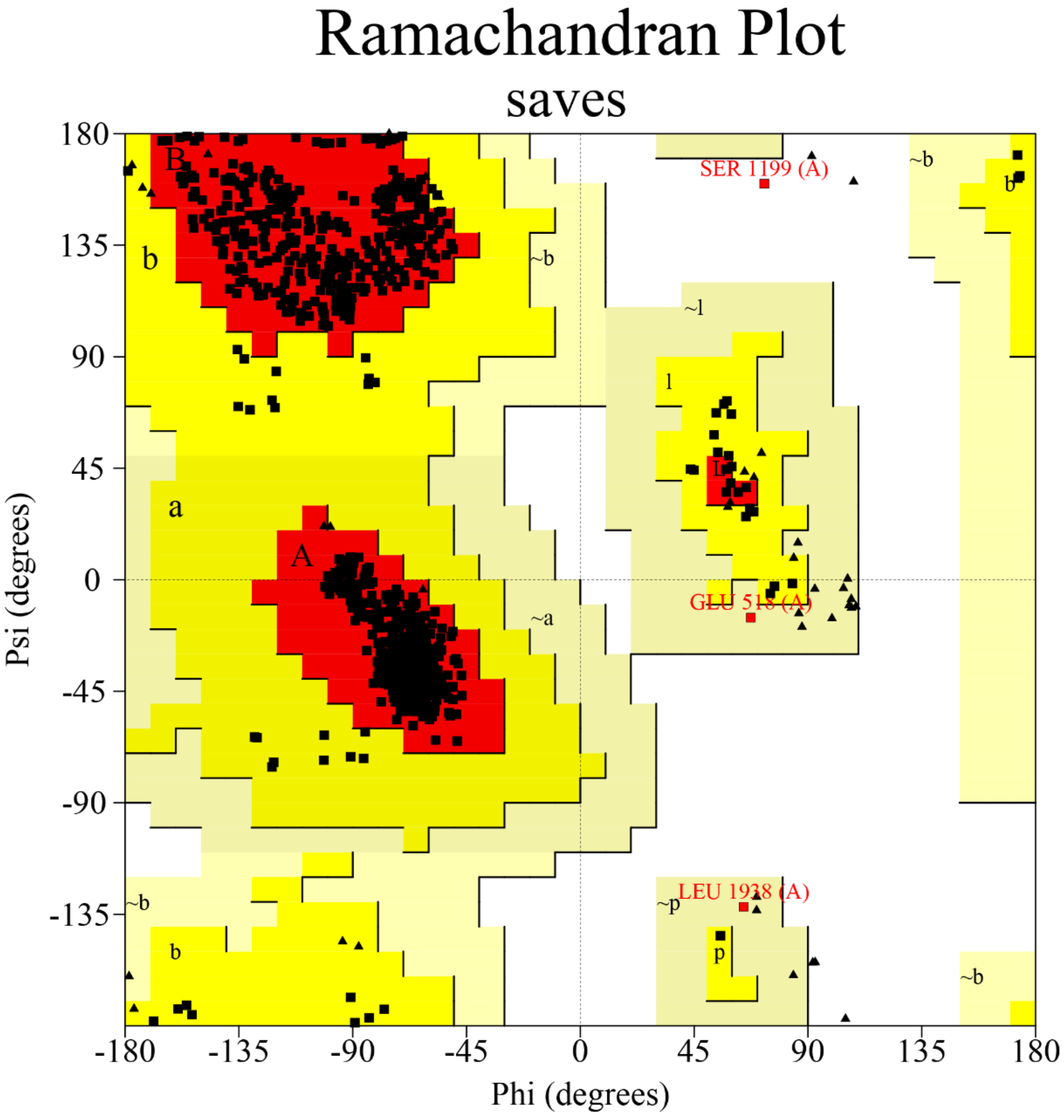

Plot statistics

Residues in most favoured regions [A,B,L]	1036	95.7%
Residues in additional allowed regions [a,b,l,p]	44	4.1%
Residues in generously allowed regions [~a,~b,~l,~p]	2	0.2%
Residues in disallowed regions	1	0.1%
	----	------
Number of non-glycine and non-proline residues	1083	100.0%
Number of end-residues (excl. Gly and Pro)	23	
Number of glycine residues (shown as triangles)	49	
Number of proline residues	50	

Total number of residues	1205	

Based on an analysis of 118 structures of resolution of at least 2.0 Angstroms and R-factor no greater than 20%, a good quality model would be expected to have over 90% in the most favoured regions.

Figure S18. Ramachandran plot for 4YKN template.

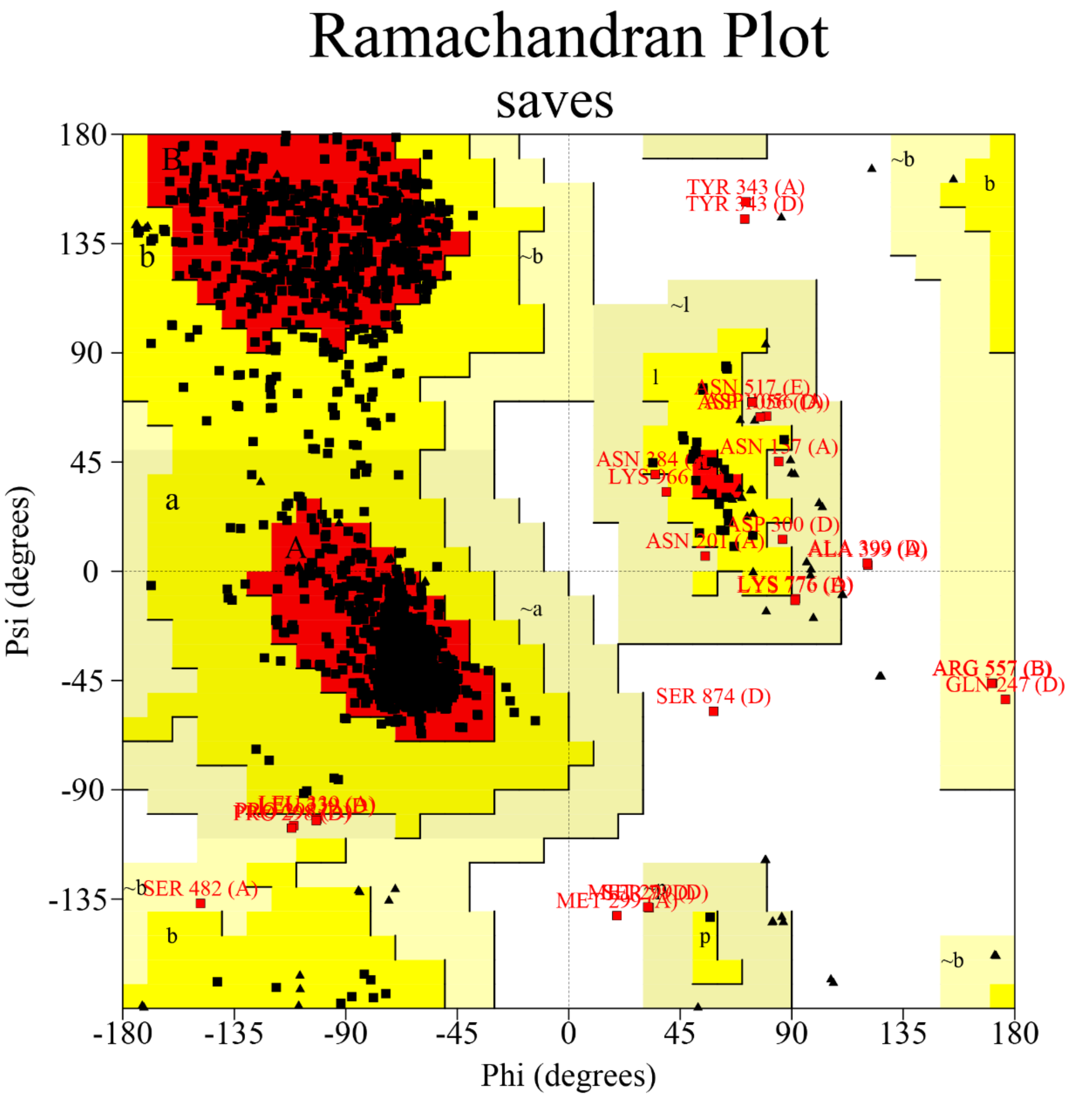

Plot statistics

Residues in most favoured regions [A,B,L]	1808	90.2%
Residues in additional allowed regions [a,b,l,p]	173	8.6%
Residues in generously allowed regions [~a,~b,~l,~p]	18	0.9%
Residues in disallowed regions	6	0.3%
	----	------
Number of non-glycine and non-proline residues	2005	100.0%
Number of end-residues (excl. Gly and Pro)	31	
Number of glycine residues (shown as triangles)	78	
Number of proline residues	102	

Total number of residues	2216	

Based on an analysis of 118 structures of resolution of at least 2.0 Angstroms and R-factor no greater than 20%, a good quality model would be expected to have over 90% in the most favoured regions.

Figure S19. Ramachandran plot for 5DXH template.

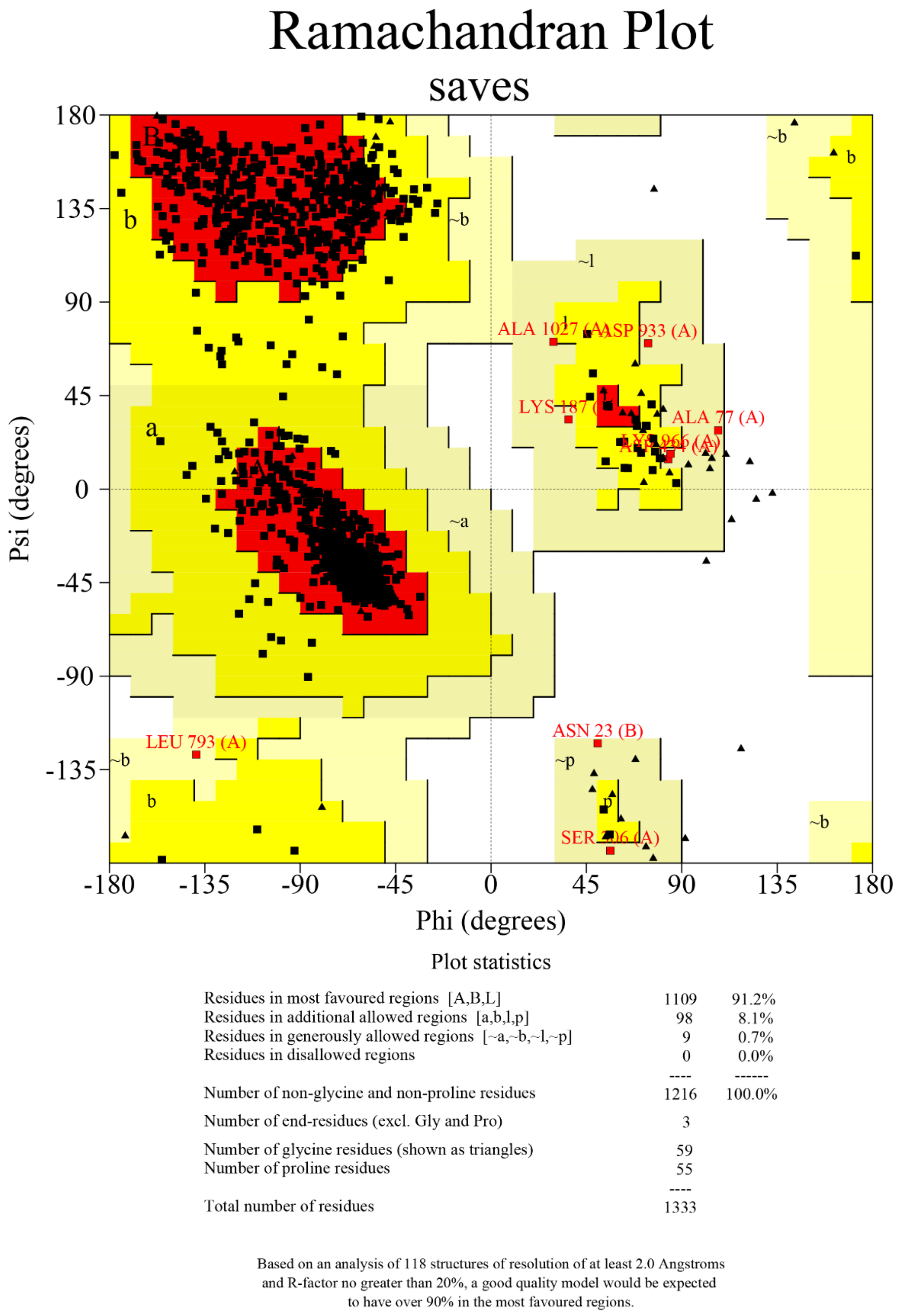

Figure S20. Ramachandran plot for the model refined by MDS.

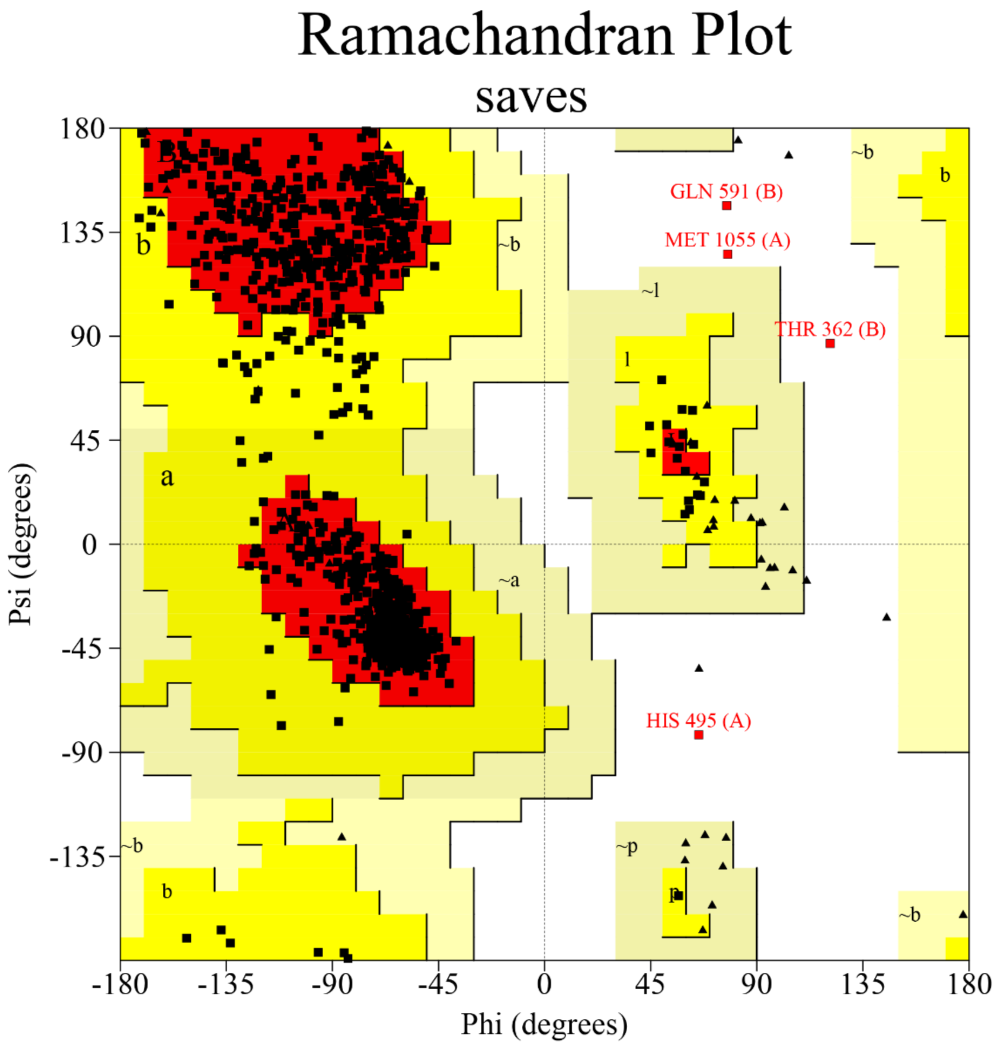

Plot statistics

Residues in most favoured regions [A,B,L]	1022	92.6%
Residues in additional allowed regions [a,b,l,p]	78	7.1%
Residues in generously allowed regions [~a,~b,~l,~p]	0	0.0%
Residues in disallowed regions	4	0.4%
	----	------
Number of non-glycine and non-proline residues	1104	100.0%
Number of end-residues (excl. Gly and Pro)	18	
Number of glycine residues (shown as triangles)	51	
Number of proline residues	53	

Total number of residues	1226	

Based on an analysis of 118 structures of resolution of at least 2.0 Angstroms and R-factor no greater than 20%, a good quality model would be expected to have over 90% in the most favoured regions.

Figure S21. Ramachandran plot for 4JPS template.

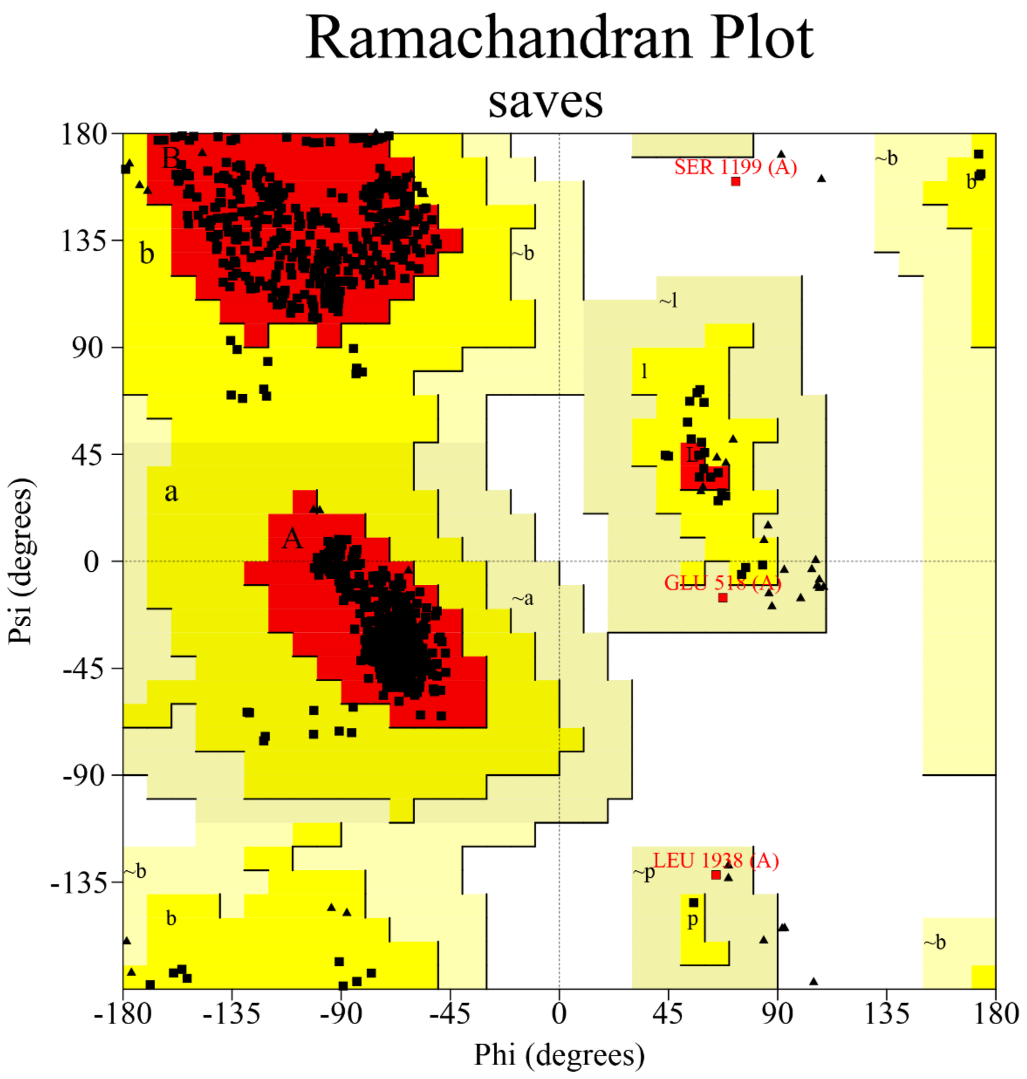

Plot statistics

Residues in most favoured regions [A,B,L]	1036	95.7%
Residues in additional allowed regions [a,b,l,p]	44	4.1%
Residues in generously allowed regions [~a,~b,~l,~p]	2	0.2%
Residues in disallowed regions	1	0.1%
	----	------
Number of non-glycine and non-proline residues	1083	100.0%
Number of end-residues (excl. Gly and Pro)	23	
Number of glycine residues (shown as triangles)	49	
Number of proline residues	50	

Total number of residues	1205	

Based on an analysis of 118 structures of resolution of at least 2.0 Angstroms and R-factor no greater than 20%, a good quality model would be expected to have over 90% in the most favoured regions.

Figure S22. Ramachandran plot for 4KYN template.

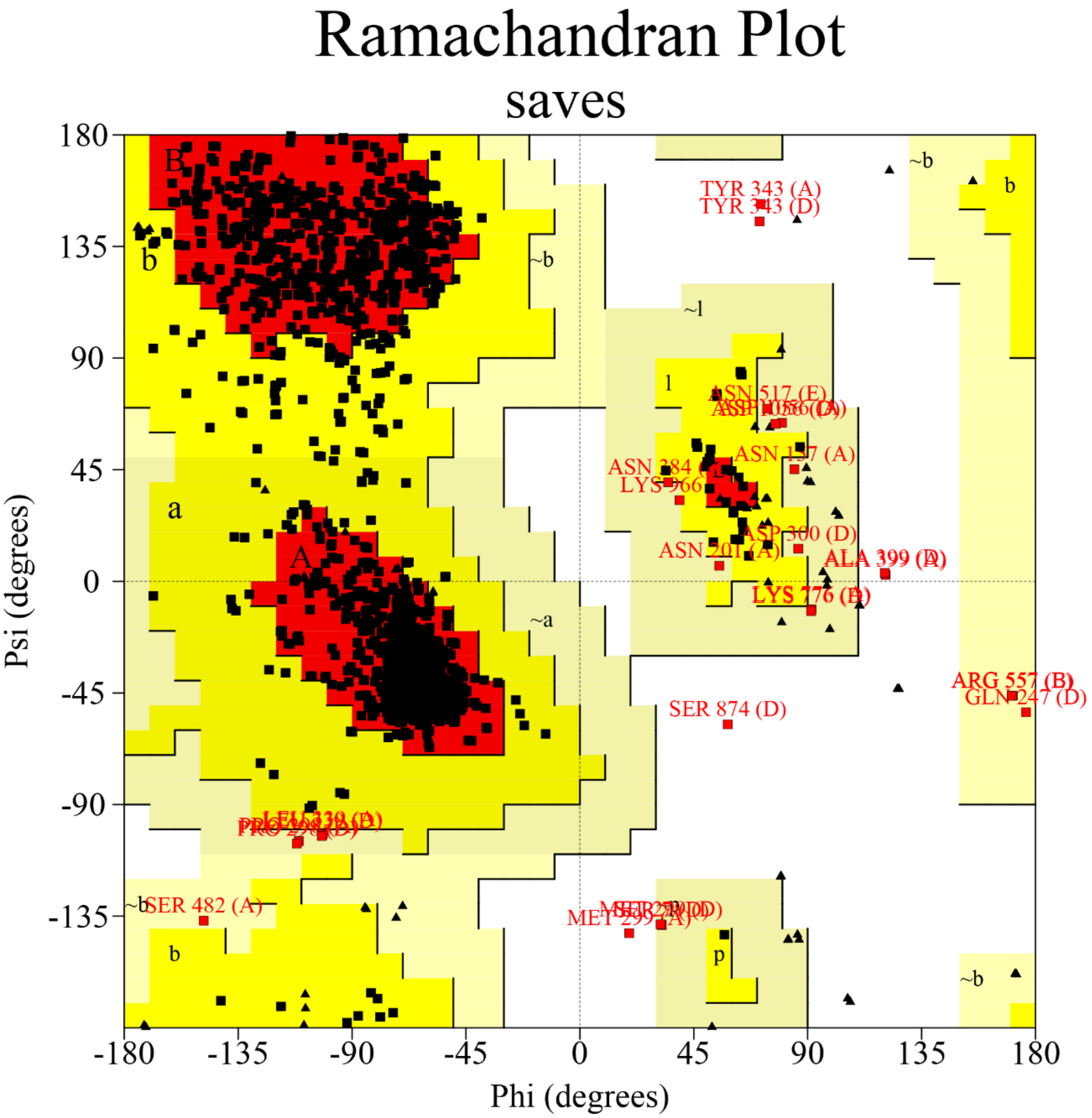

Plot statistics

Residues in most favoured regions [A,B,L]	1808	90.2%
Residues in additional allowed regions [a,b,l,p]	173	8.6%
Residues in generously allowed regions [~a,~b,~l,~p]	18	0.9%
Residues in disallowed regions	6	0.3%
	----	------
Number of non-glycine and non-proline residues	2005	100.0%
Number of end-residues (excl. Gly and Pro)	31	
Number of glycine residues (shown as triangles)	78	
Number of proline residues	102	

Total number of residues	2216	

Based on an analysis of 118 structures of resolution of at least 2.0 Angstroms and R-factor no greater than 20%, a good quality model would be expected to have over 90% in the most favoured regions.

Figure S23. Ramachandran plot for 5DXH template.

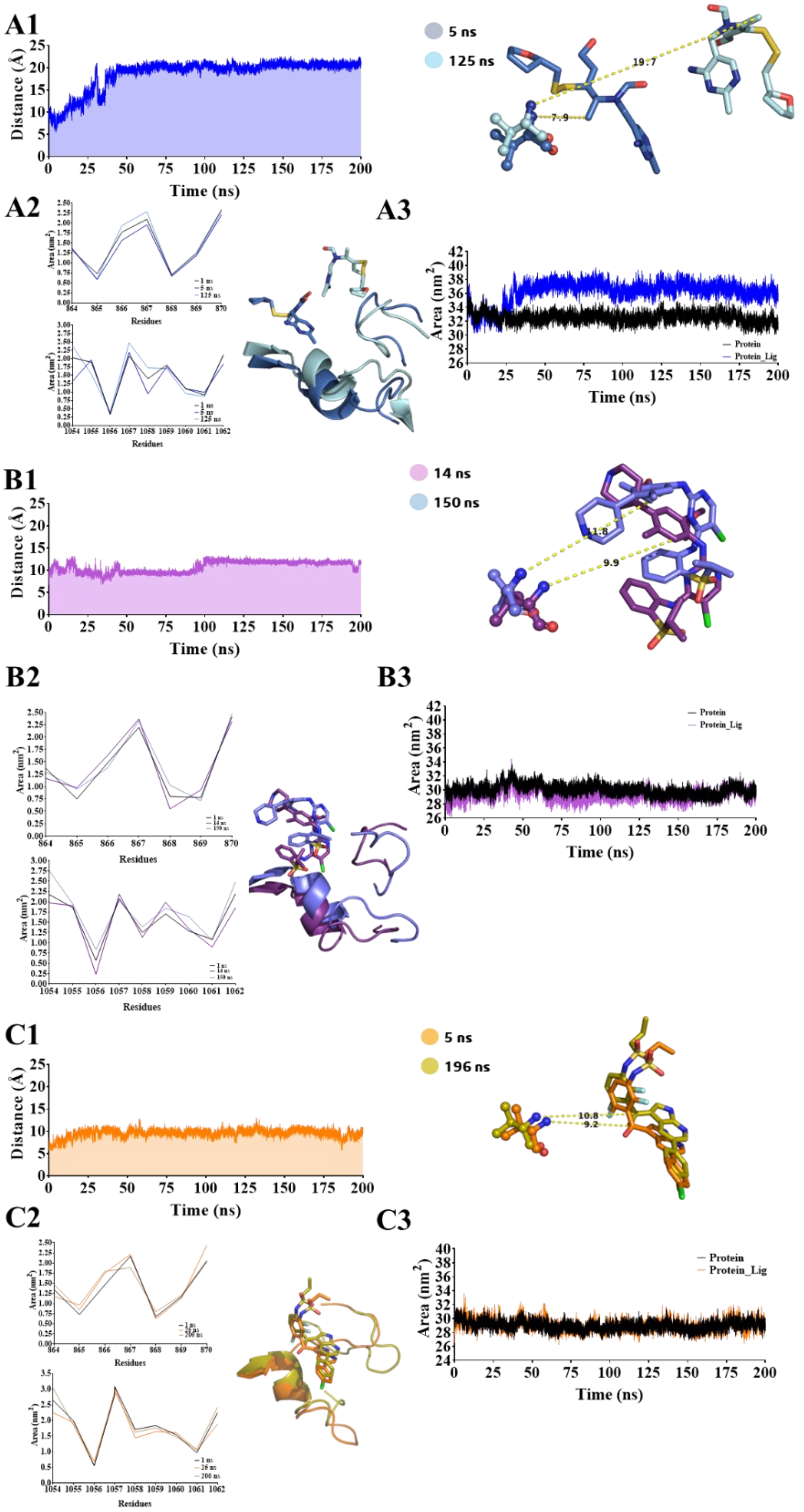

Figure S24. Loop-1 (dark blue, purple, or orange color, 851–870) and loop-2 (light blue, purple, or orange color, 1054–1062) in the presence of (**A1**) **FUR**, (**B1**) **CER** and (**C1**) **VEM** in two time periods. Variation in the volume (nm^2) of loop-1 and loop-2 related to the presence of (**A2**) **FUR**, (**B2**) **CER**, and (**C2**) **VEM**. Average solvent accessible surface area (SASA) of residues 770–932, comprising the catalytic site of the PI3Kα kinase domain of (**A3**) **FUR**, (**B3**) **CER**, and (**C3**) **VEM**.

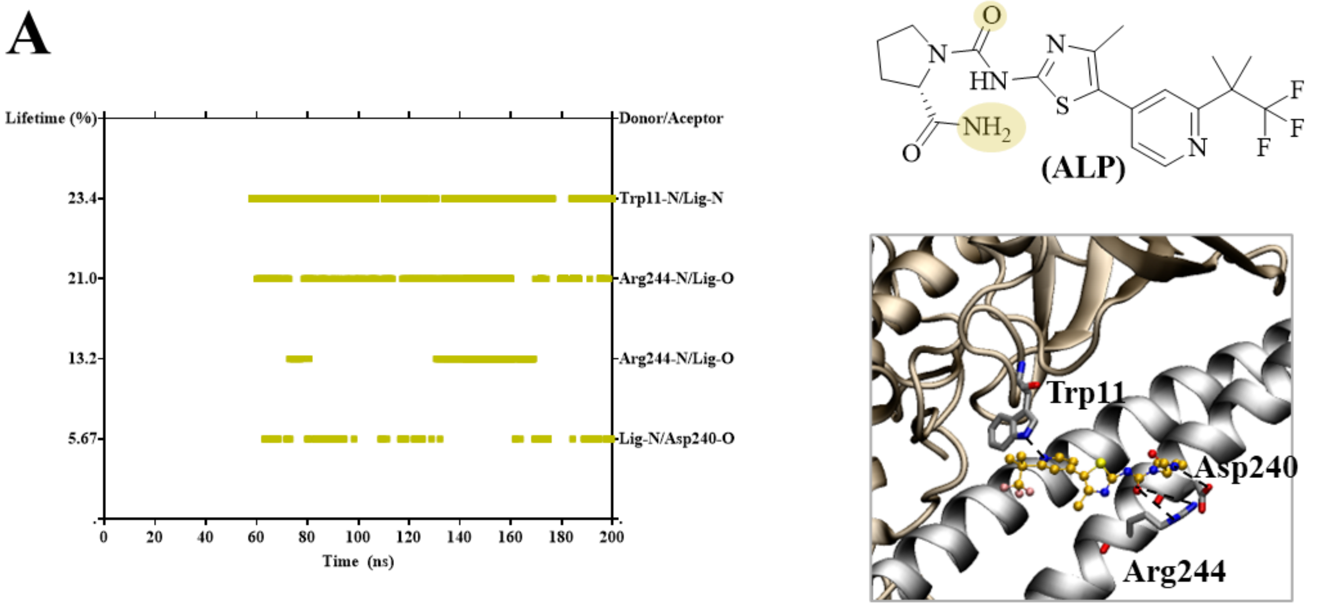

Figure S25. H-bond lifetime (%) and representative interactions of **ALP**-PI3Kα complex during 200 ns of MDS. The colored circles indicated the atoms from ligands in interaction.

Table S1. SAVES (v.6.0) results (ERRAT, 3D-1D score, and Verify 3D) for the 3D structures of the PDB templates (4JPS, 4YKN, and 5DXH) and the model refined by MDS.

Structures	ERRAT	3D-1D score <= 0.1 (%)	Verify 3D
**4JPS**	96.09	78.71	Fail
**4YKN**	99.98	74.23	Fail
**5DXH**	94.57	77.66	Fail
**MDS model**	94.84	79.60	Fail
